# From prodrug to pro-prodrug: hypoxia-sensitive antibody–drug conjugates

**DOI:** 10.1038/s41392-021-00833-8

**Published:** 2022-01-21

**Authors:** Yanming Wang, Dian Xiao, Jiaguo Li, Shiyong Fan, Fei Xie, Wu Zhong, Xinbo Zhou, Song Li

**Affiliations:** 1 National Engineering Research Center for the Emergency Drug, Beijing Institute of Pharmacology and Toxicology, 100850 Beijing, China; 2 Institute of Basic Medicine and Cancer (IBMC), Chinese Academy of Sciences, 310022 Hangzhou, Zhejiang China

**Keywords:** Drug delivery, Drug development, Drug development

**Dear Editor**,

Antibody‒drug conjugates (ADCs), famous as biological targeting prodrugs, are gradually revolutionizing clinical cancer therapy. However, less than 1% of the dosed ADCs accumulate in the tumors.^1^ Therefore, the nonspecific release of the highly toxic payload (MMAE et al., 10^−12^–10^−10^ M) is a real threat, which could induce severe off-target toxicity.^2^ This danger necessitates strict requirements for the design of the linker. To date, the mainstream enzyme cleavable linkers, including *β-*glucuronidase cleavable linkers, sulfatase-cleavable linkers, and the most popular cathepsin cleavable linkers (valine-citrulline linker), all face this nonspecific release problem,^3^ because their cleaving enzymes are widely distributed with no significant difference in their quantities between tumor tissues and normal tissues.

Hypoxia, as the most important feature of the solid tumor microenvironment, has always been a target of drug discovery programs.^4^ Hypoxia can lead to the upregulation of nitroreductase (NTR), which has been detected at levels 100–1000-fold higher than that in normoxic cells.^5^ The most important function of NTR is to reduce the nitro group to an amino group under hypoxia rather than normoxia.^4^

In this work, a novel ADC design strategy was proposed. Based on the traditional antibody targeting the “prodrug”, we further utilized hypoxia-specific drug release to design an ADC as a “pro-prodrug”, achieving a superior therapeutic index (Fig. 1a). More specifically, in tumor tissues (O_2_% < 1%), the novel nitroaromatic-containing linker was activated by the NTR to smoothly release the active drug. Notably, the beauty of this strategy is that in normal tissues (O_2_% > 10%), the pro-prodrug was metabolized and stably remained in the low toxicity and low permeability prodrug form (Cys-linker-payload), which was crucial to reduce off-target toxicity.

**Fig. 1:**
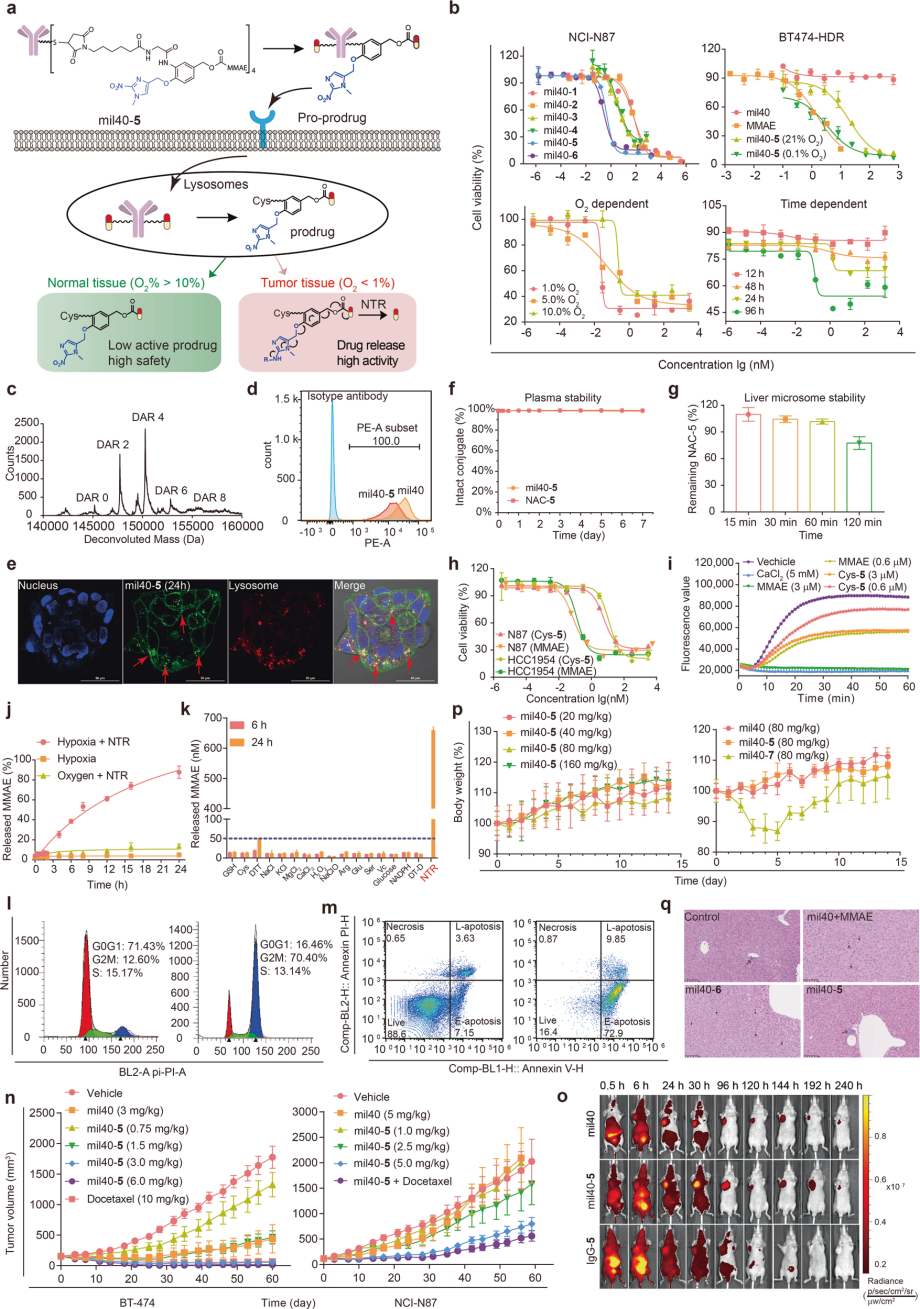
Development of novel hypoxia-sensitive ADCs for cancer therapy. **a** Structure and mechanism of optimal hypoxia-sensitive ADC. **b** In vitro cytotoxicity of mil40-**1**, mil40-**2**, mil40-**3**, mil40-**4**, mil40-**5**, and mil40-**6** in the NCI-N87 cell line and Herceptin-resistant BT-474 cell line under hypoxic conditions (0.1% O_2_). In vitro cytotoxicity of mil40-**5** under different oxygen concentrations and different hypoxia culture times in the BT-474 cell line. Data = mean ± SD (*n* ≥ 2). **c** Liquid chromatography-mass spectrometry analysis of mil40-**5. d** Flow cytometric analysis of mil40 and mil40-**5** binding to the HER2 antigens of BT-474. **e** Receptor-mediated internalization of the ADC mil40-**5** by the HER2-positive breast cancer cell line BT-474, scale bar = 50 μm. **f** Plasma stability of mil40-**5** at 37 °C. Data = mean ± SD (*n* ≥ 3). **g** Liver microsome stability of NAC-**5** under normal oxygen conditions. Data = mean ± SD (*n* ≥ 2). **h** Cytotoxicity of Cys-**5** and MMAE in HCC1954 and NCI-N87 tumor cell lines. Data = mean ± SD (n ≥ 2). **i** Inhibition test of microtubule polymerization, MMAE and CaCl_2_ were included as controls (*n* ≥ 3). **j** Drug release characteristics of NAC-**5** at the enzyme level under hypoxia without NTR, hypoxia with NTR and oxygen with NTR. Data = mean ± SD (*n* ≥ 3). **k** NTR specificity of NAC-**5** among common physiological ionic conditions. Data = mean ± SD (*n* ≥ 2). **l** Cell cycle analysis of mil40-**5** at concentrations of 0 nM and 10 nM in BT-474 cells (*n* ≥ 2). **m** Apoptosis analysis of mil40-**5** at concentrations of 0 nM and 10 nM in BT-474 cells (*n* ≥ 2). **n** Therapy experiment with mil40-**5** in BT-474 and NCI-N87 subcutaneous tumor-bearing mice. Female mice xenografted with BT-474 cells and NCI-N87 cells were treated with mil40, mil40-**5**, or mil40-**5** combined with docetaxel on days 0, 7, 14, and 21 (*n* = 6/group). Mil40-**5** (2.5 mg/kg) and docetaxel (8 mg/kg) were administered once a week in the combination group. **o** Fluorescence imaging of the naked antibody and ADC in an NCI-N87 xenograft model (*n* = 5/group). **p** Changes in the body weights of CD-1 mice in the tolerance test. Male CD-1 mice were injected with mil40-**5** and mil40-**7** via the tail vein (*n* = 3/group). **q** Histopathological studies of ADC mil40-**5** in CD-1 mice. The mice were dosed at 20 mg/kg, and histopathological analysis was performed on the seventh day after administration (*n* = 3/group). The magnification of each picture is ×20

Two classical hypoxia-sensitive groups, *p*-nitrobenzyl and nitroimidazole, were chosen to develop *p*-nitrobenzyl-based linkers (**1**, **2**, **3**, and **4**) and a nitroimidazole-based linker (**5**). Corresponding ADCs (mil40-**1**/**2**/**3**/**4**/**5**) were generated by conjugating monomethyl auristatin E (MMAE) to an anti-HER2 antibody (mil40) through the above linkers (Supplementary Fig. S1). Traditional valine-citrulline-ADC (mil40-**6**, Supplementary Fig. S1) and valine-alanine-ADC (mil40-**7**, Fig. S1) were also synthesized as controls.

To quickly verify their potency, hypoxia-sensitive ADCs were evaluated for their cytotoxicity against the HER2 + cell lines NCI-N87, BT-474, and HCC1954 under hypoxia (0.1% O_2_, Fig. 1b, Supplementary Fig. S2). Excitingly, mil40-**5** exhibited a significantly higher activity than other ADCs. The EC_50_ values were between 0.00012 nM and 0.46 nM, which were similar to that of the control ADC (mil40-**6**, Table S1). Mil40-**5** was also confirmed to be HER2-specific, with low activity against HER2− cell lines (EC_50_ > 100 nM, Supplementary Fig. S2). Only 5.8%, 18.5%, 56.1%, 62.7% of MMAE released from *p*-nitrobenzyl-based linkers explained the poor efficacies of ADCs mil40-**1**/**2**/**3**/**4**, (Supplementary Fig. S3a, b, c). The generation and characterization of the optimal ADC mil40-**5** are shown in the supplementary materials (Supplementary Fig. S4a, b, c, d, e). Liquid chromatography-mass spectrometry analysis showed an average drug-to-antibody ratio (DAR) of 4.0 for mil40-**5** (Fig. 1c). Flow cytometry analysis and laser confocal microscopy revealed binding and subsequent endocytosis into lysosomes (Pearson correlation coefficient = 0.918, Fig. 1d, e).

The hypoxia cytotoxicity ratio (HCR) is the gold standard for hypoxia-sensitive drugs. Significantly, mil40-**5** showed oxygen concentration-dependent cytotoxicity (Fig. 1b). In Herceptin-sensitive cells, the EC_50_ value under hypoxia (1% O_2_, EC_50_ = 0.025 nM) was 10 times lower than that under normoxia (10% O_2_, EC_50_ = 0.23 nM). In Herceptin-resistant cells, the HCR further increased, reaching as much as 27 (0.73 nM vs. 19.48 nM), owing to the shielding of antibody activity. For highly toxic payloads, this ten-fold difference was important for improving the therapeutic index of ADCs. Additionally, the maximum inhibition rate increased with prolonged hypoxia culture time, which provided further evidence for hypoxia activation of mil40-**5** (Fig. 1b).

Under normoxia, mil40-**5** was metabolized and stably existed as the prodrug Cys-**5** (21% O_2_, Supplementary Fig. S5a). Both mil40-**5** and *N*-acetyl-*l*-cysteine (NAC)-**5** exhibited excellent stability in plasma and under physiological ionic conditions, with less than 2% of MMAE released (Fig. 1f, Fig. S5b). Furthermore, NAC-**5** demonstrated good liver microsome stability with 77% unchanged (Fig. 1g) and no MMAE released (Supplementary Fig. S5c), indicating reduced hepatotoxicity. As designed, Cys-**5** significantly reduced cytotoxicity by 51–78 times compared with MMAE (Fig. 1h). This was due to the decreased tubulin inhibitory activity of Cys-**5** compared to MMAE, with EC_50_ values of 8.784 μM and 1.259 μM (Fig. 1i, Fig. S5d). Interestingly, the efficacy of Cys-**5** under hypoxia was also not enhanced (Supplementary Fig. S5e), which was due to the limited permeability of Cys-**5** (MlogP = −1.818, Supplementary Table S2). The above results suggested that mil40-**5** could reduce off-target toxicity in normal tissues through the stable, low toxicity prodrug Cys-**5** (Supplementary Fig. S5f).

Under hypoxia, mil40-**5** smoothly released the active drug MMAE in BT-474 and NCI-N87 cells (0.1% O_2_, Supplementary Fig. S6a). More precisely, approximately 90% MMAE was effectively released within 24 h under hypoxia with NTR, which was far more than that in normoxia and hypoxia without NTR (<10%, Fig. 1j). Meanwhile, other common physiological ions did not lead to the significant release of free MMAE, which verified the NTR specificity of the linker (Fig. 1k). MMAE induced cell cycle arrest in the G2/M phase and eventually led to apoptosis. When treated with mil40-**5** (10 nM, 0.1% O_2_), the percentage of BT-474 cells in the G2/M phase increased from 10.78 to 82.75%, and the apoptosis percentage increased from 10.78 to 82.75% (Fig. 1l, m). Similar trends were also observed in NCI-N87 cells (Supplementary Fig. S6b). The above results suggested that mil40-**5** exerted cytotoxicity through the hypoxia-NTR-specific release of MMAE (Supplementary Fig. S6c).

Encouraged by the in vitro results, the therapeutic potential of the preferred mil40-**5** was then determined in BT-474 and NCI-N87 mouse models. In BT-474 mouse model, mil40-**5** exhibited better efficacy than mil40 combined with MMAE (Supplementary Fig. S7a), and showed significant tumor growth inhibition at 6 mg/kg (*p* < 0.0001, Fig. 1n). More importantly, four doses of 6 mg/kg mil40-**5** induced tumor regression in five out of six mice, exhibiting better efficacy than docetaxel at 10 mg/kg (*p* = 0.0012, Supplementary Fig. S7a). In the NCI-N87 mouse model, mil40-**5** also exhibited better efficacy than mil40 at the dose of 5 mg/kg (*p* = 0.009, Fig. 1n). In addition, mil40-**5** was expected to achieve better efficacy through combined use of docetaxel (*p* = 0.0007, Supplementary Fig. S7b). Then, the targeting effect in vivo was confirmed by in vivo imaging analysis. The fluorescence-labeled mil40 and mil40-**5** were initially visualized in the tumor sites within 24 h, and the tumor-located image was clearly maintained over 10 days (Fig. 1o).

Finally, we explored the safety advantages of mil40-**5** in CD-1 mice. The maximum tolerable dose (MTD) of mil40-**5** was not reached even at the extremely high dosage of 160 mg/kg, and it showed no obvious signs of toxicity or body weight loss (Fig. 1p, Supplementary Fig. S8a). In comparison, mice in the control ADC (mil40-**7**) group began to experience sustained weight loss (0–5 days) at 80 mg/kg (Fig. 1p). When tested at a treatment dose (20 mg/kg), mice in the mil40-**5** group did not show significant tissue lesions. However, an additional increase in hepatocyte mitoses was observed in H&E staining of the liver and grain-red ratio in the control ADC (mil40-**6**) and mil40+MMAE groups, indicating potential hepatotoxicity (Fig. 1q, Supplementary Fig. S8b).

In conclusion, the first hypoxia-sensitive ADC was developed as a pro-prodrug. It was based on a novel nitroimidazole-based linker, realizing oxygen-dependent cytotoxicity. A 27-fold cytotoxicity enhancement was observed when the ADC was activated by hypoxia. This controllable cytotoxicity further transformed into significant efficacy and a superior therapeutic index in vivo. A significantly higher MTD (>160 mg/kg) was obtained compared with traditional ADC (<80 mg/kg). This novel strategy could be universally applied to existing payloads and different antibodies, which represent next-generation ADCs.

## Supplementary information

Supplementary Materials for From prodrug to pro-prodrug: hypoxia-sensitive antibody–drug conjugates

## Data Availability

All relevant data are available in Supplementary Information and from the authors.
