# Supplementary material for: From prodrug to pro-prodrug: hypoxia-sensitive antibody–drug conjugates
Source: Signal Transduct Target Ther. 2022 Jan 21;7:20. doi: 10.1038/s41392-021-00833-8 (PMC8776858; doi:10.1038/s41392-021-00833-8)
Supplement: Supplementary file 1 — Supplementary Materials for From prodrug to pro-prodrug: hypoxia-sensitive antibody–drug conjugates [file 41392_2021_833_MOESM1_ESM.docx]

**Supplementary Materials for**

**From prodrug to pro-prodrug: Hypoxia sensitive antibody-drug conjugates**

Yanming Wang^1‡^, Dian Xiao^1‡^, Jiaguo Li^2‡^, Shiyong Fan^1^, Fei Xie^1^, Wu Zhong^1*^, and Xinbo Zhou^1*^ and Song Li^1^

^1^National Engineering Research Center for the Emergency Drug, Beijing Institute of Pharmacology and Toxicology, Beijing 100850, China.

^2^Institute of Basic Medicine and Cancer (IBMC), Chinese Academy of Sciences, Hangzhou, Zhejiang 310022, China

^‡^Authors contributed equally

Corresponding author: [zhongwu@bmi.ac.cn](mailto:zhongwu@bmi.ac.cn) (W.Z.), [zhouxinbo@bmi.ac.cn](mailto:zhouxinbo@bmi.ac.cn) (X.Z.)

**This file includes:**

Supplementary Figure 1-8.

Supplementary Table 1-2.

Experimental section.

**Fig. S1.** Structures of *p*-nitrobenzyl based hypoxia-sensitive ADCs (mil40-**1**/**2**/**3**/**4**) and traditional dipeptide-based ADCs (mil40-**6**/**7**).


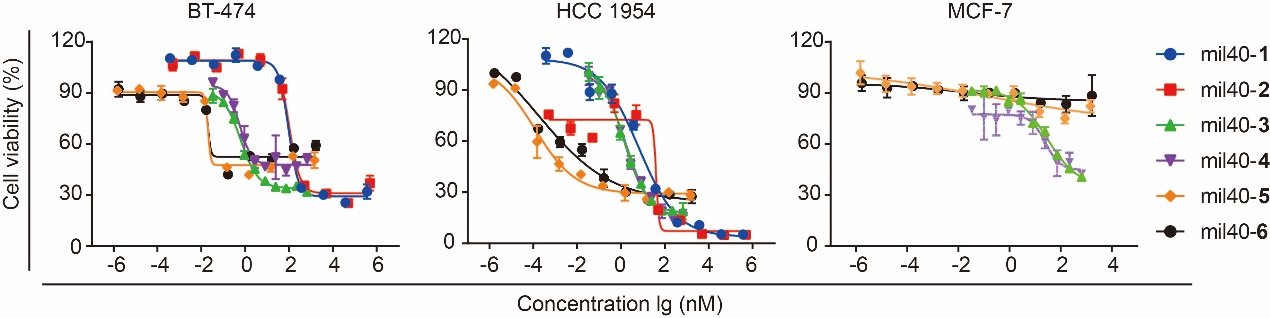


**Fig. S2.** *In vitro* cytotoxicity of mil40-**1**/**2**/**3**/**4**/**5/6** in HER2+ cell lines BT-474, HCC1954 and HER2- cell line MCF-7. Data = mean ± SD (n ≥ 2).


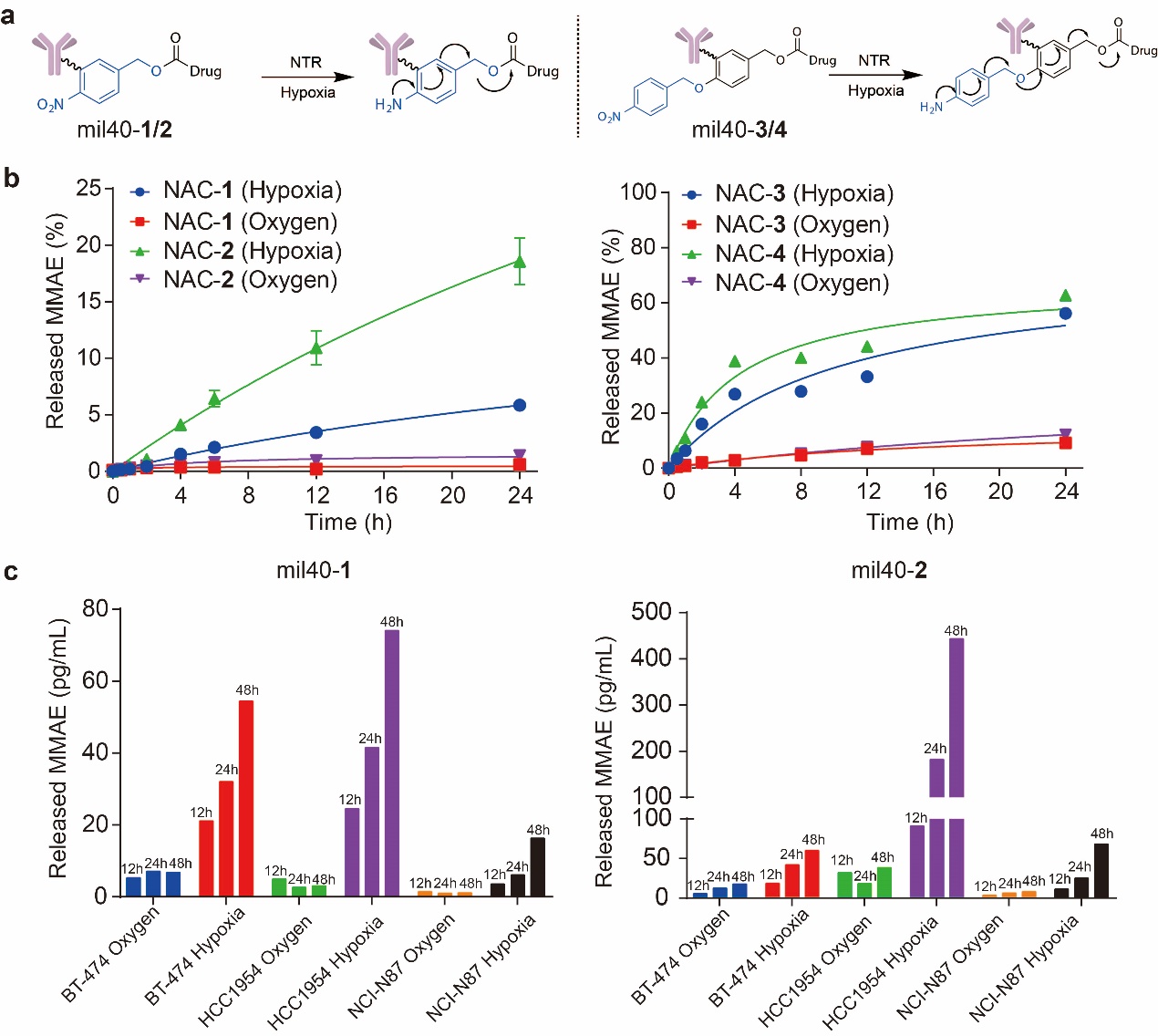


**Fig. S3**. Drug release test of the *p*-nitrobenzyl based ADCs. **a** Proposed drug release mechanism of the *p*-nitrobenzyl based ADCs. **b** The drug release characteristics of NAC-**1/2/3/4** at enzyme level under hypoxia with NTR. Data = mean ± SD (n ≥ 3). **c** The drug release characteristics of mil40-**1**/**2** in HER2+ cell lines BT-474, HCC1954, NCI-N87 at 12 h, 24h and 48 h.


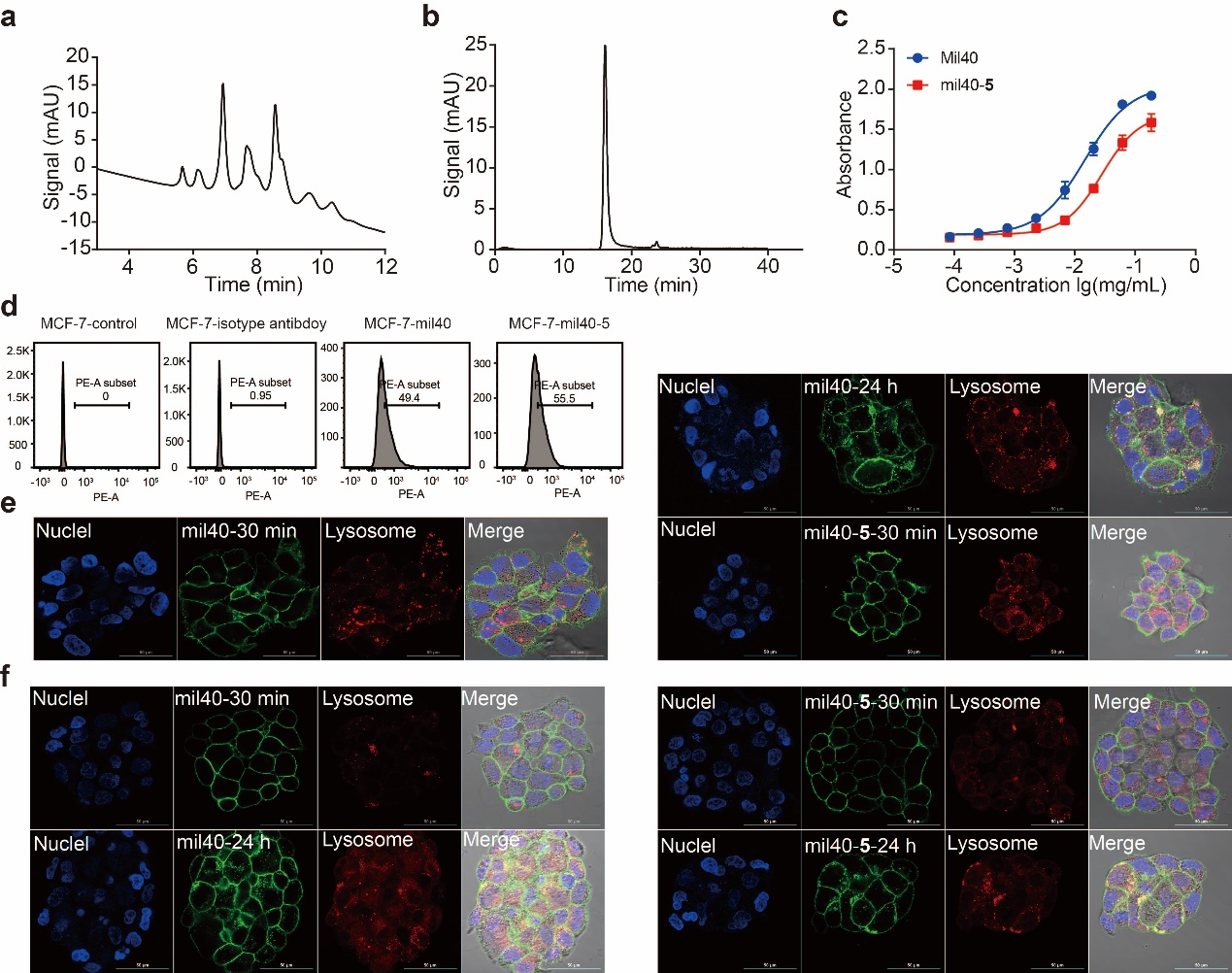


**Fig. S4.** Quality control of optimal ADC mil40-**5**. **a** Hydrophobic interaction chromatography (HIC) analysis of mil40-**5**. **b** Size exclusion chromatography (SEC) analysis of mil40-**5**. **c** Measuring the relative affinities of mil40 and mil40-**5** to HER2 antigens. Data = mean ± SD (n ≥ 2). **d** Mil40 and mil40-**5** binding to the Her2 antigens of HER2- cells MCF-7. **e** Receptor-mediated binding and internalization of mil40-**5** by the HER2+ breast cancer cell line BT-474, scale bar = 50 μm. **f** Receptor-mediated binding and internalization of mil40-**5** by the HER2+ breast cancer cell line BT474-HDR, scale bar = 50 μm.


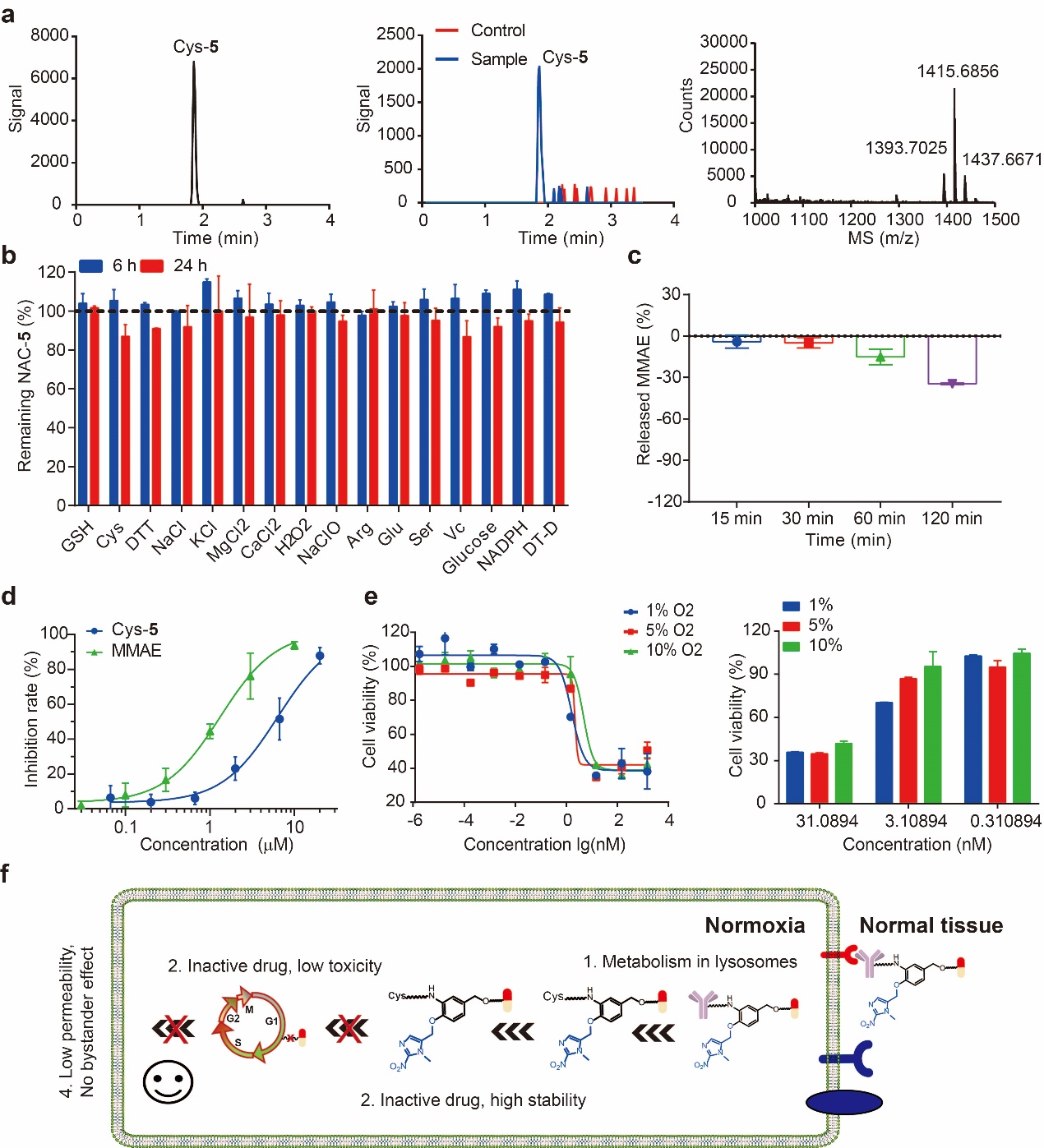


**Fig. S5.** Study of hypoxia-activated ADC under normoxia. **a** Detection of normoxic metabolite Cys-**5** in BT-474 cell lines. **b** Multiple chemical factors on the stability of NAC-**5** under normal oxygen condition at 6 h and 24 h. Data = mean ± SD (n ≥ 2). **c** Released MMAE in liver microsome stability test. Data = mean ± SD (n ≥ 2). **d** Tubulin inhibitory activity of Cys-**5** and MMAE. Data = mean ± SD (n ≥ 3) **e** Cytotoxicity of Cys-**5** and MMAE under different oxygen concentrations in BT-474 cells. Data = mean ± SD (n ≥ 2). **f** Cell process and mechanism of hypoxia-activated ADC mil40-**5** under normoxia.


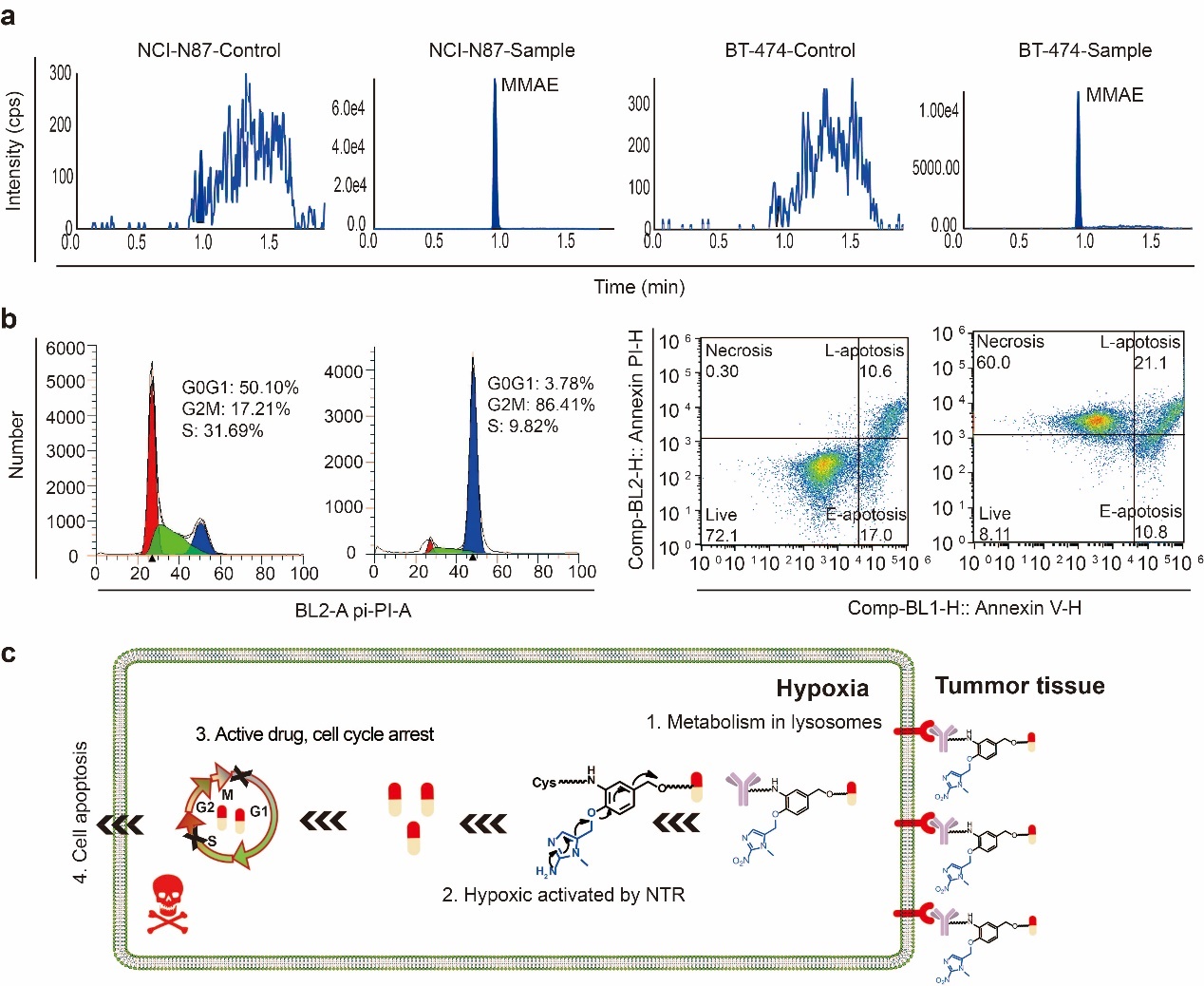


**Fig. S6.** Study of hypoxia-activated ADC under hypoxia. **a** Detection of MMAE HER2+ BT-474 and NCI-N87 cells (0.1% O_2_). **b** Cell-cycle and apoptosis analysis of mil40-**5** under the concentration of 0 nM and 10 nM in NCI-N87 cells (0.1% O_2_, n ≥ 2). **c** Cell process and mechanism of hypoxia-activated ADC mil40-**5** under normoxia.


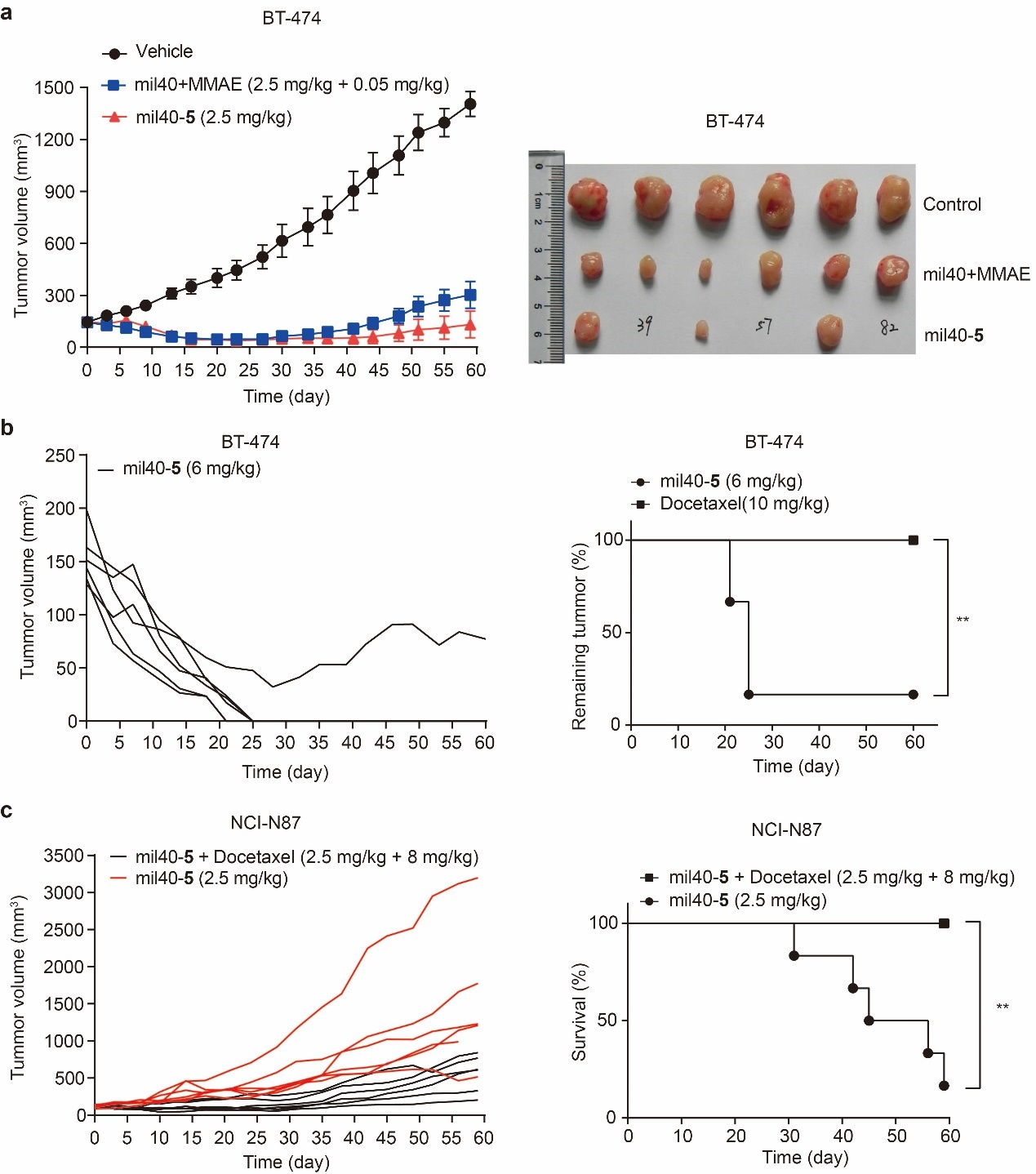


**Fig. S7.** *In vivo* efficacy evaluation of mil40-**5** in BT-474 and NCI-N87 models. **a** Therapy experiment with mil40-**5** in BT-474 subcutaneous tumor-bearing mice. Female mice xenografted with BT-474 cells were treated with mil40-**5** and mil40-**5** combined with MMAE on days 0, 7, 14, and 21 (n = 6/group). 2.5 mg/kg mil40-**5** and 0.05 mg/kg MMAE were administered in the combination group. **b** Tumor volumes are shown for individual animals treated with mil40-**5**. And remaining tumor percent are shown for mil40-**5** and docetaxel group. **c** Tumor volumes are shown for individual animals treated with mil40-**5** and combination group. When the tumor volume exceeded 1000 mm^3^, the mice were treated as dead. The survival percent are shown for mil40-**5** and combination group.


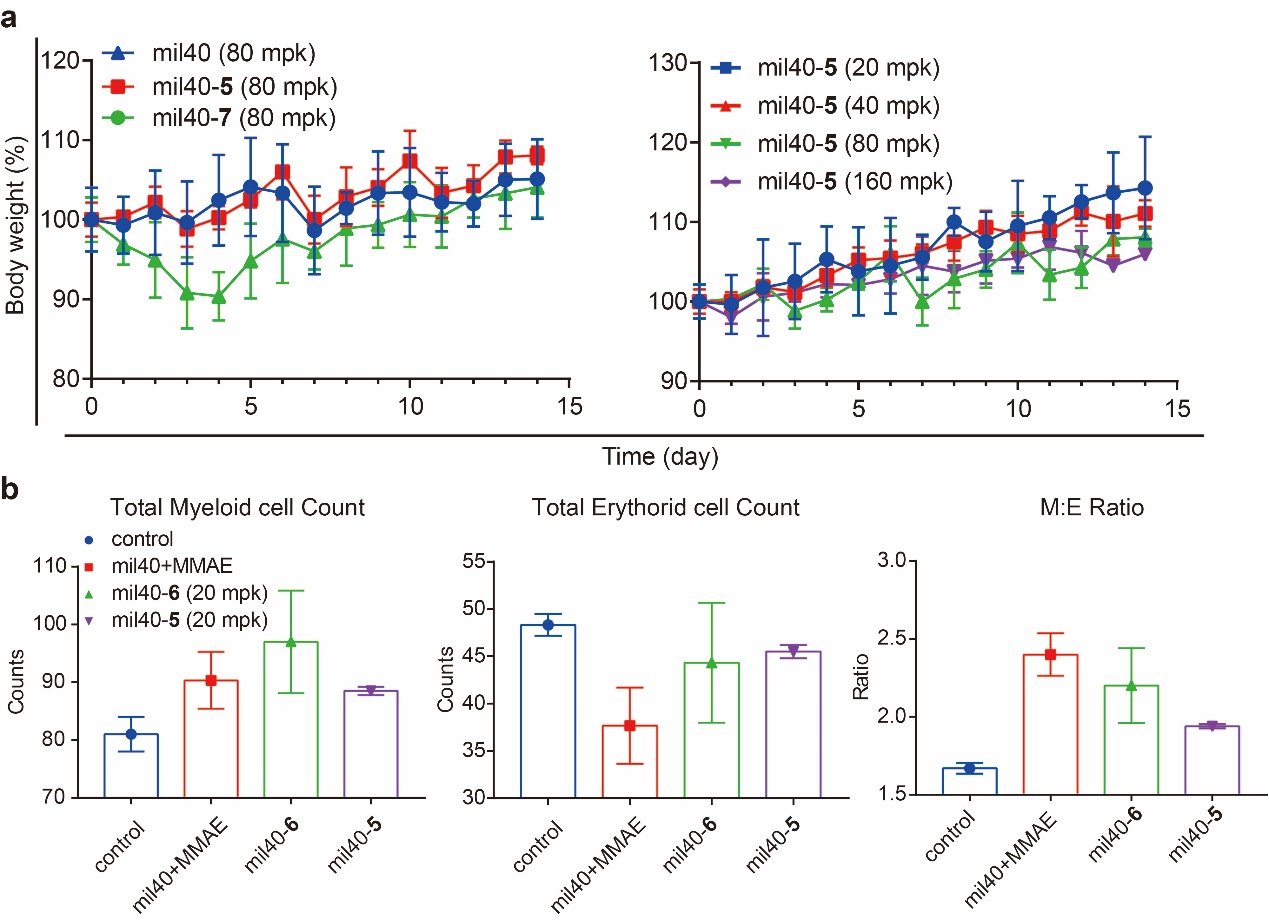


**Fig. S8.** *In vivo* safety evaluation of hypoxia-activated ADC. **a** Changes in body weights of the CD-1 mice in the tolerance test, male CD-1 mice were injected with mil40-**5** and mil40-**7** via the tail vein (n = 3/group). **b** Histopathological studies of ADC mil40-**5** and mil40-**6** in CD-1 mice, the mice were dosed at 20 mg/kg (n = 3/group).

**Table S1.** *In vitro* activity of the ADCs and cytotoxic agents.

| Cell lines | Test compounds (EC_50_ nM) | | | | | |
| --- | --- | --- | --- | --- | --- | --- |
|  | mil40-**3** | mil40-**4** | mil40-**5** | mil40-**6** | mil40 | MMAE |
| BT-474 | 0.6146 | 0.5623 | 0.01992 | 0.01991 | 0.6637 | 0.03386 |
| HCC1954 | 1.347 | 1.67 | 0.0001169 | 0.0001767 | >100 | 0.07509 |
| NCI-N87 | 3.34 | 3.199 | 0.4598 | 0.2696 | 0.6286 | 0.1297 |
| MCF-7 | 29.1 | 25.05 | >100 | >100 | >100 | 12.44 |

**Table S2.** Permeability prediction of Cys-linker-MMAE and MMAE

| Compounds | MlogP | S+Peff  (cm/s×10^4^) | S+MDCK  (cm/s×10^7^) | Perm_Cornea  (cm/s×10^7^) |
| --- | --- | --- | --- | --- |
| Cys-**5** | -1.818 | 0.075 | 6.215 | 12.46 |
| MMAE | 1.191 | 0.353 | 21.818 | 63.09 |

MlogP: moriguchi model of octanol-water partition coefficient, larger logP values indicate higher lipophilicity; S+Peff: human effective jejunal permeability, larger S+Peff values indicate greater permeability and the predicted value for membrane permeable molecules is usually > 0.25; S+MDCK: apparent MDCK COS permeability, larger S+MDCK values also indicate greater permeability and the predicted value for membrane permeable molecules is usually > 20; Perm_Cornea: permeability through rabbit cornea, larger Perm_Cornea values indicate greater membrane permeability.

1. Experimental section

1.1 Generation of the NTR-based antibody-drug conjugates

Humanized anti-HER2 antibody mil40 in *L*-Histidine buffer (20 mM, pH ≈ 7.5) was treated with tris(2-carboxyethyl)phosphine hydrochlorides (~2.3 equivalents) for 90 min. To the reduced antibody was added the maleimide derivatives (8 equivalents) in dimethylacetamide. After 1–2 h, the reactions were quenched with NAC (8 equivalents). The mixture was adjusted to weak acidity (pH ≈ 5.5) with diluted AcOH (0.3 mol/L) before buffer exchange by elution through Sephadex G25, and concentrated by centrifugal ultrafiltration.^1^ The concentrations of the generated ADCs were determined by UV absorption at 280 nm. The final ADCs solutions were sterile filtered through a 0.2-μm filter under sterile conditions and stored at -80 °C before use for analysis and testing.

1.2 The stability assays of ADC in plasma

The human plasma (LOT#: BRH1343165) was purchased from BioreclamationIVT (Westbury, NY, USA). The NAC (900 μL, 0.41 mg/mL, pH 7.4) was added to the solution of compound **5** (100μL, 70.9 μg/mL) and incubated in water bath at 37 °C; HPLC inspection revealed complete conversion to NAC-**5** conjugates after 10 minutes, and the reaction mixture was mixed into an equal volume of human plasma and incubated in a incubator at 37 °C.^2^ Aliquots were collected at subsequent time points and quenched with cold acetonitrile before frozen at -80 °C. After sampling is completed, all samples were melted at room temperature and centrifuged, and analyzed by LC-MS/MS. Results were based on the AUC of the off-target MMAE at each time point. Analogously, The ADC mil40-**5** in PBS (2.0 mg/mL) was mixed into an equal volume of human plasma and incubated in a sterile incubator at 37 °C, and the aliquots were collected and detection using the methods described above.

1.3 Nitroreductase reactivity

To the phosphate buffer saline (390 μL, 100mM, pH 7.4) was filled with nitrogen and added to NADPH (75 μL, 20 mM), NTR (Sigma: E.C. 1.6.2.4, #CYP004; 10 μL, 3 mg/mL), the mixture is incubated at 37 °C for 10 min and added the stock solution of linker-MMAE conjugates (NAC-**1**, NAC-**2**, NAC-**5**),^3^ then continue to incubate at 37 °C under hypoxia (0.1% O_2_). Aliquots were collected at each subsequent time points and quenched with acetonitrile before frozen at -80 °C. After sampling is completed, all samples were melted at room temperature and centrifuged to remove protein, and analyzed by LC-MS/MS.

1.4 The specificity of nitroreductase

The NAC-**5** in phosphate buffer saline (PBS) was added to the solution of NADPH (500 μM) + NTR (0.5 μM) under hypoxia, and added to the solution of glutathione (GSH, 1 mM), cysteine (Cys, 1 mM), dithiothreitol (DTT, 1 mM), NaCl (10 mM), KCl (150 mM), MgCl_2_ (2.5 mM), CaCl_2_ (2.5 mM), H_2_O_2_ (10 μM), NaClO (10 μM), arginine (Arg, 1 mM), (Glu, 1 mM), (Ser, 1 mM), ascorbic acid (Vc, 1 mM), glucose (10 mM), NADPH (500 μM), DT-diaphorase (0.5 μM), NTR (0.5 μM) under normal oxygen.^4-8^ The above mixed solution were incubated at 37 °C for 24 h, and then the NAC-**5** and the released MMAE were detected simultaneously by LC-MS/MS.

1.5 Cell-level drug release assay

The HER2-positive cell lines NCI-N87 and BT-474 were all purchased from American Type Culture Collection (ATCC). After the cells were added to the ADCs, changed medium which contain corrsponding test compound after 24 hours under hypoxia (0.1% O_2_).^9^ Discard the culture medium of NCI-N87 and BT-474, and cells were detached with trypsin/EDTA and inactivate trypsin by adding excess serum containing medium. Centrifuged and washed cell pellets twice with ice cold PBS, then, extracted cell pellets by adding cold methanol, and the suspensions were kept at -20 °C for 30 min before centrifuged at 13000 g. Supernatants were evaporated by nitrogen gas blowing, and the resulting residues were redissolved in methanol which contains internal standard (IS=100 nM Alprazolam). The released MMAE in samples were analyzed by LC-MS/MS.

1.6 Evaluation of ADCs for tumor cell killing *in vitro*

The HER2-positive cell line HCC1954 and the HER2-negative cell line MCF-7, MDA-MB-468, and MDA-MB-231 were also purchased from ATCC. The oxygen concentration in the cell culture environment were controlled by Galaxy^®^ CO_2_ Incubator (48 R, #CO48312044, Eppendorf, Saxony, Germany). Cells (3.3 × 10^4^ cells/mL) were added to each well of a 96-well plate after which 10 μL compound was also added to the assay plate. The plate was incubated for 7 days at 37 °C, 5% CO_2_, 95% humidity. Then, the plates were incubated at room temperature for about 10 min, 40 μL CTG reagent was added to each well. Luminescence was detected using the EnSpire Plate Reader, and Prism5 for Windows (Graphpad software, Inc., La Jolla, CA, USA) was used for data analysis.

1.7 Confocal analysis for intracellular localization

The lysosomal traffcking of mil40-**5** was examined with reference to a published method.^2^ Briefly, BT-474 cells were seeded at a density of 2×10^4^ cells/well and incubated at 37 °C overnight. Then, the cells were treated with 5 μg/mL mil40 or mil40-**5** for 30 minutes at 4 °C or 16 h at 37 °C. After washing the wells with ice-cold PBS, the cells were fixed with 4% paraformaldehyde for 10 min and permeabilized with 0.2% Triton X-100 for 5 min. The mil40 and mil40-**5** present were detected with an Alexa Fluor 488-labeled goat anti-human IgG antibody. The lysosomes were labeled with a lysosomal-associated membrane protein 1 (LAMP-1) antibody followed by an Alexa Fluor 568-labeled goat anti-mouse IgG (H + L). Fluorescence images were obtained using a Nikon A1 confocal microscope (Nikon, Corp., Tokyo, Japan).

1.8 Flow cytometry for cell cycle arrest analysis

The HER2-positive cell lines BT-474, and NCI-N87 were seeded at a density of 1×10^6^ cells/well and exposed to mil40-**5** with various concentrations (0, 1, and 10 μg/mL) for 24 h, and allowed to incorporate bromodeoxyuridine (BrdUrd; Beyotime Biotechnology, Shanghai, China) for 20 min. Nascent DNA synthesis was detected with anti-BrdUrd FITC, and total DNA content was detected with PI. The cell cycle position and apoptosis analyses were measured by a FACSCalibur (BD Biosciences, Franklin Lake, NJ, USA).

1.9 Evaluation of ADCs for anti-tumor efficacy in xenograft tumor models

All *in vivo* experiments were conducted in accordance with the Institutional Animal Care and Use Committee in a facility fully accredited by the Association for Assessment and Accreditation of Laboratory Animal Care (APU number: ON-CELL-XEN-06012020). Female NOD/SCID mice 6–8 weeks of age (~ 22 g), were inoculated subcutaneously with 1×10^7^ BT-474 breast tumor cells in 0.2 mL DMEM-Matrigel mixture (1:1 ratio) for tumor development. Analogously, BALB/c nude mice were inoculated subcutaneously with 5×10^6^ NCI-N87 gastric cancer cells in serum-free RPMI1640 medium. The treatment started when the mean tumor size reached approximately 150 mm^3^, and the animals were given ADC (mil40-**5**) , antibody (mil40), and vehicle on days 0, 7, 14, and 21. The animals were monitored twice weekly for tumor size and body weight. Tumor volume was calculated using the formula: TV = a × b^2^/2, where “a” and “b” are long and short diameters of a tumor, respectively. All animal experiments were performed by following the protocol approved by the Institutional Animal Care and Use Committee at Pharmaron Co., Ltd.

1.10 *In vivo* fluorescence imaging of tumor xenograft

The *in vivo* fluorescence imaging experiments were investigated using a NCI-N87 Balb/c-nude mice xenograft model.^1^ The naked antibody mil40, the ADC mil40-**5** and the IgG-**5** were labeled with DyLight 680 according to the manufacturer’s instructions (Dylight 680 Antibody Labeling Kit, Thermo Fisher Scientific). When the tumor size reached approximately 300 mm^3^, mice in the three groups were injected via the tail veins with the dose of 10 mg/kg. The mice were imaged under anesthesia at the indicated time points after the injection using the IVIS^®^ Lumina XRMS SeriesⅢ imaging system (Waltham, MA, USA).

1.11 High-dose tolerance test

The CD-1 mice in 7–9 weeks of age were purchased from Beijing Vital River Laboratory Animal Technology Co., Ltd. Each dose test group consisted of 6 animals (3 males and 3 females) and all animals were adapted for at least 3 days prior to dosing. After administration via the tail vein with ADCs, all test animals monitored body weight changes and observed behaviors once a day. After the last observation, all surviving animals will be euthanized by inhalation of 90%–100% CO_2_.

1.12 Hematology and histopathology

The CD-1 mice in 7–9 weeks of age (n=9/group) were dosed via the tail vein with 20 mg/kg ADCs (mil40-**5** and mil40-**6**), the mixture of mil40+MMAE (equivalent dose or volume to 20 mg/kg mil40-**5**), or vehicle. After administration, all animals were subjected to whole-blood hematological analysis on on days 4, 7, and 14. During the test, half of the test animals were euthanized to execute the histopathologically testing on day 7. The organs mainly included the liver and lung, tissue samples were subjected to paraffin embedding, sectioning and H&E staining. Similarly, the tested animal bones were taken from the femur to make two bone marrow smears, which were observed under the microscope together with the histopathological sections after the trial.

1.13 Statistical analysis

Data are expressed as means ± SD. For comparison of the above indicators, unpaired two-tailed *t* test for multiple comparisons was used. The level of significance was set at *p* < 0.05. Statistical analyses were performed using Prism 8 for Windows (Graphpad software, Inc., La Jolla, CA, USA).

1.14 Synthetic Procedures

*6-(2,5-dioxo-2,5-dihydro-1H-pyrrol-1-yl)hexanoic acid* (**S3**)

Maleic anhydride (4.86 g, 49.55 mmol) was added to a solution of the 6-aminohexanoic acid (5.0 g, 38.11 mmol) in AcOH (150 mL). The mixture was stirred at 120 °C for 6 h. The reaction mixture was poured into water after cooling to room temperature (rt) and extracted with ethyl acetate (3 × 20 mL). The organic layers were combined, washed with brine, dried over anhydrous Na_2_SO_4_, and evaporated under reduced pressure to give the crude product. Purification was performed by silica column chromatography in 1:6 ethyl acetate/petroleum ether (v/v). A white solid was obtained (5.92 g), 74% yield as a white solid; ^1^H-NMR (400 MHz, DMSO-*d*_6_): *δ* 11.98 (br, 1H), 7.01 (s, 2H), 3.39 (m, 2H), 2.17 (t, *J* = 7.4 Hz, 2H), 1.51 - 1.44 (m, 2H), 1.24 - 1.17 (m, 2H). ESI-MS m/z (M-H)^−^ calculated for C_7_H_7_NO_4_ 210.1; found 210.0.

*2,5-dioxopyrrolidin-1-yl 6-(2,5-dioxo-2,5-dihydro-1H-pyrrol-1-yl)hexanoate* (**S4**)

A solution of **S3** (4.66 g, 22.0 mmol), *2,4,6*-trimethylpyridine (11.6 mL, 88.0 mmol,) and *N*-Hydroxysuccinimide (5.08 g, 44.0 mmol) in THF (100 mL) was cooled to 0 °C; the trifluoroacetic anhydride (6.12 mL, 44.0 mmol) was added dropwise over 30 min. The reaction mixture was stirred at rt for 1 h, and then evaporated under reduced pressure and re-dissolved in ethyl acetate (200 mL). The organic layers was washed with 1N HCl solution and brine, dried over anhydrous Na_2_SO_4_, and evaporated under reduced pressure to give the crude product. Purification was performed by silica column chromatography in 1:2 ethyl acetate/petroleum ether (v/v) to give 4.71 g (69%) of **S4** as a colorless oil, low temperature placed and converted to a white solid. ^1^H-NMR (400 MHz, CDCl_3_): *δ* 6.69 (s, 2H), 3.53 (t, *J* = 7.3 Hz, 2H), 2.84 (s, 4H), 2.61 (t, *J* = 7.4 Hz, 2H), 1.78 (m, 2H), 1.63 (m, 2H), 1.45 - 1.37 (m, 2H). ESI-MS m/z (M+H)^+^ calculated for C_14_H_17_N_2_O_6_ 309.1, found 309.4; ESI-MS m/z (M+Na)^+^ calculated for C_14_H_16_N_2_NaO_6_ 331.1, found 331.2.

*2,5-dioxopyrrolidin-1-yl(1r,4r)-4-((2,5-dioxo-2,5-dihydro-1H-pyrrol-1-yl)methyl)cyclohexane-1-carboxylate* (**S7**)

A solution of **S5** (7.86 g, 50.0 mmol), **S1** (4.90 g, 50.0 mmol) in AcOH (100 mL) was stirred at 120 °C for 6 h. The reaction mixture was poured into water after cooling to rt and extracted with ethyl acetate (3 × 50 mL). The organic layers were combined, washed with brine, dried over anhydrous Na_2_SO_4_, and evaporated under reduced pressure to give the crude product as a white solid (9.96 g, 84% yield), which was carried out directly without purification. A solution of the obtained white solid (13.1 g, 55 mmol), *2,4,6*-trimethylpyridine (26.4 mL, 200 mmol) and *N*-Hydroxysuccinimide (23.0 g, 200 mmol) in THF (250 mL) was cooled to 0 °C; the trifluoroacetic anhydride (27.8 mL, 200 mmol) was added dropwise over 45 min. The reaction mixture was stirred at rt for 18 h, and then chloroform (300 mL) and a HCl solution (1 N, 250 mL) were added to the reaction mixture, and then extracted with DCM (3 ×100 mL). The organic layer obtained by extraction was washed twice with HCl (1 N, 250 mL). The filtrate after drying over anhydrous sodium sulfate was concentrated under vacuum to give a crude yellow solid. The solid was beaten with diethyl ether (3 x 200 mL) to give a white powdery solid (15 g, 90% yield). ^1^H-NMR (400 MHz, DMSO-*d*_6_): *δ* 6.71 (s, 1H), 3.39 (d, *J* = 7.2 Hz, 2H), 2.82 (d, *J* = 7.3 Hz, 4H), 2.58 (m, 1H), 2.15 (m, 2H), 1.80 (m, 2H), 1.56 (m, 1H), 1.54 (m, 2H), 1.06 (m, 2H). ESI-MS m/z (M+NH_4_)^+^ calculated for C_16_H_22_N_3_O_6_ 352.2, found 352.6; ESI-MS m/z (M+Na)^+^ calculated for C_16_H_18_N_2_NaO_6_ 357.1, found 357.4.

*methyl 5-methyl-2-nitrobenzoate* (**S9**)

A solution of **S8** (1.0 g, 5.52 mmol) in MeOH (30 mL) was added to concentrated H_2_SO_4_ and stirred at 75 °C for 18 h, and then evaporated under reduced pressure to give the crude product. Purification was performed by silica column chromatography to give 920 mg (75% yield) of **S9** as a white solid. ^1^H-NMR (400 MHz, DMSO-*d*_6_): *δ* 8.00 (d, *J* = 8.4 Hz, 1H), 7.66 (d, *J* = 1.4 Hz, 1H), 7.61 (dd, *J* = 8.4 Hz, 1H), 3.84 (s, 3H), 2.45 (s, 3H). ESI-MS m/z (M+H)^+^ calculated for C_9_H_10_NO_4_ 196.1, found 196.0; ESI-MS m/z (M+NH_4_)^+^ calculated for C_9_H_13_N_2_O_4_ 213.1, found 213.0.

*methyl 5-(bromomethyl)-2-nitrobenzoate* (**S10**)

To a solution of **S9** (1.0 g, 5.1 mmol), AIBN (catalyst, 50 mg), and *N*-Bromo Succinimide (1.0 g, 5.6 mmol) in benzene (50 mL) was heated to reflux and stirred for 18 h. After the reaction stops, evaporated the solvent under reduced pressure to give a crude product, further purification was performed by silica column chromatography to give 1.14 g (81% yield) of **S10** as a white solid. ^1^H-NMR (400 MHz, CDCl_3_): *δ* 7.91 (d, *J* = 8.4 Hz, 1H), 7.75 (d, *J* = 2.0 Hz, 1H), 7.64 (dd, *J* = 8.4 Hz, 1H), 5.50 (s, 2H), 3.94 (s, 1H). ESI-MS m/z (M+H)^+^ calculated for C_9_H_9_BrNO_4_ 272.9, found 272.9; ESI-MS m/z (M+NH_4_)^+^ calculated for C_9_H_12_BrN_2_O_4_ 292.1, found 292.9.

*5-(hydroxymethyl)-2-nitrobenzoic acid* (**S11**)

To a solution of **S10** (250 mg, 0.91 mmol) of a mixed solution of acetone and water (10 mL, 1:1 v/v) was added to Na_2_CO_3_ (0.48 g, 4.55 mmol) and heated to 65 °C and stirred for 6 h, then additional NaOH (0.18 g, 5 eq) was added and reflux was continued for 1 h. After the reaction stops, the acetone was concentrated and removed, and adjusted the pH of the solution was to 2 with concentrated HCl, and extracted with EA multiple times. The organic phases were combined and washed three times with saturated NaCl and dried over MgSO_4_. Purification was performed by silica column chromatography to give 140 mg (76% yield) of **S11** as a reddish brown flake solid. ^1^H-NMR (400 MHz, DMSO-*d*_6_): *δ* 7.97 (d, *J* = 8.4 Hz, 1H), 7.74 (d, *J* = 2.0 Hz, 1H), 7.65 (dd, *J* = 8.4 Hz, 1H), 5.59 (s, 1H), 4.63 (d, *J* = 3.18.4 Hz, 2H). ESI-MS m/z (M-H)^-^ calculated for C_8_H_6_NO_5_ 196.0, found 195.9.

*tert-butyl (2-(5-(hydroxymethyl)-2-nitrobenzamido)ethyl)carbamate* (**S12**)

A solution of **S11** (500 mg, 2.53 mmol), EDCI (0.73 g, 3.8 mmol), HOBt (0.51 g, 3.8 mmol), and DIPEA (0.49 g, 3.8 mmol) in DMF (15 mL) was stired at rt for 1 h, and then added the corresponding amine (0.81 g, 5.06 mmol). The mixture was stirred at rt for 18 h, and evaporated under reduced pressure to give the crude product. Purification was performed by silica column chromatography to give 0.76 g (89% yield) of **S12** as a yellow gelatinous solid. ^1^H-NMR (400 MHz, DMSO-*d*_6_): *δ* 8.66 (t, *J* = 5.6 Hz, 1H), 8.02 (d, *J* = 8.4 Hz, 1H), 7.59 (d, *J* = 8.4 Hz, 1H), 7.53 (s, 1H), 6.88 (t, *J* = 11.5 Hz, 1H), 5.59 (t, *J* = 5.6 Hz, 1H), 4.62 (d, *J* = 5.6 Hz, 2H), 3.23 (q, *J* = 6.2 Hz, 2H), 3.09 (q, *J* = 6.2 Hz, 2H), 1.39 (s, 9H). ESI-MS m/z (M+H)^+^ calculated for C_15_H_22_N_3_O_6_ 340.2, found 340.2; ESI-MS m/z (M+Na)^+^ calculated for C_15_H_21_N_3_NaO_6_ 362.1, found 362.2.

*N-(2-aminoethyl)-5-(hydroxymethyl)-2-nitrobenzamide* (**S13**)

A solution of **S12** (760 mg, 2.24 mmol) in 2N HCl/EA (3 mL) was stired at rt overnight. The solvent was removed under reduced pressure to give the crude product, and purification was performed by silica column chromatography to give 0.58 g (94% yield) of **S13** as a light yellow solid powder. ^1^H-NMR (400 MHz, DMSO-*d*_6_): *δ* 8.94 (t, *J* = 5.6 Hz, 1H), 8.04 (d, *J* = 8.4 Hz, 1H), 7.90 (br, 1H), 7.67 (s, 1H), 7.62 (d, *J* = 8.4 Hz, 1H), 5.69 (br, 1H), 4.63 (s, 2H), 3.49 (q, *J* = 5.9 Hz, 2H), 2.95 (t, *J* = 6.7 Hz, 2H). ESI-MS m/z (M+H)^+^ calculated for C_10_H_14_N_3_O_4_ 240.1, found 240.2; ESI-MS m/z (M+Na)^+^ calculated for C_10_H_13_N_3_NaO_4_ 262.1, found 262.2.

*N-(2-(6-(2,5-dioxo-2,5-dihydro-1H-pyrrol-1-yl)hexanamido)ethyl)-5-(hydroxymethyl)-2-nitrobenzamide* (**S14**)

A solution of **S13** (760 mg, 2.24 mmol) in DMF (20 mL) was added **S4** (1.18 g, 3.82 mmol) and DIPEA (0.49 g, 3.82 mmol). The mixture was stired at rt overnight and then evaporated the solvent under reduced pressure to give a crude product, further purification was performed by silica column chromatography to give 1.14 g (81% yield) of **S10** as a light yellow glassy solid. ^1^H-NMR (400 MHz, DMSO-*d*_6_): *δ* 8.67 (t, *J* = 5.6 Hz, 1H), 8.02 (d, *J* = 8.4 Hz, 1H), 7.87 (t, *J* = 5.4 Hz, 1H), 7.59 (d, *J* = 9.4 Hz, 1H), 7.52 (s, 1H), 7.00 (s, 2H), 5.61 (t, *J* = 5.7 Hz, 1H), 4.62 (d, *J* = 5.6 Hz, 2H), 3.39 (q, *J* = 7.2 Hz, 2H), 3.22 (m, 4H), 2.05 (t, *J* = 7.5 Hz, 2H), 1.48 (m, 4H), 1.18 (m, 2H). ESI-MS m/z (M+H)^+^ calculated for C_20_H_25_N_4_O_7_ 433.2, found 433.6; ESI-MS m/z (M+Na)^+^ calculated for C_20_H_24_NaN_4_O_7_ 455.2, found 455.4.

*3-((2-(6-(2,5-dioxo-2,5-dihydro-1H-pyrrol-1-yl)hexanamido)ethyl)carbamoyl)-4-nitrobenzyl (4-nitrophenyl) carbonate* (**S15**)

To a solution of **S14** (1.10 g, 2.54 mmol) in DMF (10 mL) was added bis(*4*-Nitrophenyl) carbonate (1.55 mg, 5.09 mmol) and DIPEA (0.66 g, 5.09 mmol) and then stirred at rt overnight. The reaction mixture was evaporated under reduced pressure and the purification was performed by silica column chromatography to give 1.38 g (97%) of **S15** as a light yellow solid. ^1^H-NMR (400 MHz, DMSO-*d*_6_): *δ* 8.75 (t, *J* = 5.3 Hz, 1H), 8.34 (dt, *J* = 9.2 Hz, 2H), 8.11 (d, *J* = 8.1 Hz, 1H), 7.86 (t, *J* = 5.7 Hz, 1H), 7.76 (dd, *J* = 8.3 Hz, 1H), 7.71 (d, *J* = 1.7 Hz, 1H), 7.61 (dt, *J* = 9.2 Hz, 2H), 7.00 (s, 2H), 5.44 (s, 2H), 3.35 (t, *J* = 6.6 Hz, 2H), 3.24 (m, 4H), 3.05 (t, *J* = 7.4 Hz, 2H), 1.47 (m, 4H), 1.17 (m, 2H). ESI-MS m/z (M+H)^+^ calculated for C_27_H_28_N_5_O_11_ 598.2, found 598.4; ESI-MS m/z (M+NH_4_)^+^ calculated for C_27_H_31_NaN_6_O_11_ 615.2, found 615.3.

*Linker-MMAE conjugate* (**1**)

To a solution of **S15** (73.24 mg, 0.12 mmol), MMAE (80.0 mg, 0.11 mmol), and HOBt (15.06 mg, 0.11 mmol) in DMF (6 mL) was added DIPEA (28.80 mg, 0.22 mmol). The reaction mixture was stirred at rt overnight and then poured into water (20 mL). The mixture was extracted with ethyl acetate (3 × 20 mL), the organic layers were combined, washed with brine, dried over anhydrous Na_2_SO_4_, and evaporated under reduced pressure to give the crude product. Purification was performed by silica column chromatography in 80:1-10:1 DCM/methanol (v/v) to give 83 mg (64%) of **1** as a white solid. ^1^H-NMR (400 MHz, DMSO-*d*_6_): *δ* 8.70 (br, 1H)), 8.44 (t, *J* = 8.9 Hz, 0.5H), 8.19 (d, *J* = 8.7 Hz, 0.5H), 8.04 (dd, *J* = 8.4 Hz, 1H), 7.73 - 7.84 (m, 1.5H), 7.73 - 7.57 (m, 2.5H), 7.31 - 7.15 (m, 5H), 7.00 (s, 2H), 5.44 - 5.36 (dd, *J* = 5.0 Hz, 1H), 5.31 - 5.12 (m, 2H), 4.76 - 4.65 (br, 1H), 4.54 - 4.24 (m, 3H), 4.00 (m, 2H), 3.79 (m, 0.5H), 3.58 - 3.46 (m, 1.5H), 3.38 (m, 2H), 3.24 - 3.12 (m, 12H), 2.97 - 2.81 (m, 4H), 2.41 (d, *J* = 16.0 Hz, 1H), 2.25 (m, 1H), 2.14 - 1.99 (m, 5H), 1.80 - 1.74 (br, 3H), 1.52 - 1.43 (m, 5H), 1.29 - 1.09 (m, 7H), 1.05 - 0.94 (m, 6H), 0.88 - 0.75 (m, 18H). ESI-HRMS m/z (M+Na)^+^ calculated for C_60_H_89_N_9_NaO_15_ 1198.6376, found 1198.5708; ESI-MS m/z (M+2H)^2+^ calculated for C_60_H_91_N_9_O_15_ 588.8317, found 588.7847.

*N-(2-((1r,4r)-4-((2,5-dioxo-2,5-dihydro-1H-pyrrol-1-yl)methyl)cyclohexane-1-carboxamido)ethyl)-5-(hydroxymethyl)-2-nitrobenzamide* (**S16**)

To a solution of **S13** (530 mg, 2.22 mmol) in DMF (20 mL) was added **S7** (815 mg, 2.44 mmol) and DIPEA (0.31 g, 2.44 mmol). The mixture was stired at rt overnight, and then evaporated the solvent under reduced pressure to give a crude product. Further purification was performed by silica column chromatography to give 660 mg (65% yield) of **S16** as a light yellow glassy solid. ^1^H-NMR (400 MHz, DMSO-*d*_6_): *δ* 8.65 (t, *J* = 5.6 Hz, 1H), 8.02 (d, 1H), 7.77 (t, *J* = 5.6 Hz, 1H), 7.59 (d, *J* = 9.4 Hz, 1H), 7.51 (s, 1H), 7.01 (s, 2H), 5.59 (t, *J* = 5.7 Hz, 1H), 4.62 (d, *J* = 5.6 Hz, 2H), 3.25-3.18 (m, 6H), 3.22 (m, 4H), 2.00 (tt, *J* = 3.4 Hz, 1H), 1.72 (d, *J* = 10.6 Hz, 2H), 1.62 (d, *J* = 10.6 Hz, 2H), 1.52 (m, 1H), 1.26 (qd, *J* = 4.2 Hz, 2H), 0.89 (qd, *J* = 3.4 Hz, 2H). ESI-MS m/z (M+H)^+^ calculated for C_22_H_27_N_4_O_7_ 459.2, found 459.5; ESI-MS m/z (M+Na)^+^ calculated for C_22_H_26_N_4_NaO_7_ 481.2, found 481.2.

*3-((2-((1r,4r)-4-((2,5-dioxo-2,5-dihydro-1H-pyrrol-1-yl)methyl)cyclohexane-1-carboxamido)ethyl)carbamoyl)-4-nitrobenzyl (4-nitrophenyl) carbonate* (**S17**)

To a solution of **S16** (0.46 g, 1.0 mmol) in DCM (10 mL) was added 4-nitrophenyl carbonochloridate (0.40 mg, 2 mmol) and pyridine (5 mL), and then stirred at rt overnight. The reaction mixture was evaporated under reduced pressure and the purification was performed by silica column chromatography to give 0.30 g (48%) of **S17** as a white solid. ^1^H-NMR (400 MHz, DMSO-*d*_6_): *δ* 8.75 (t, *J* = 5.2 Hz, 1H), 8.34 (d, *J* = 9.0 Hz, 2H), 8.10 (d, *J* = 8.4 Hz, 1H), 7.77 (m, 2H), 7.70 (s, 1H), 7.60 (d, *J* = 9.0 Hz, 2H), 7.00 (s, 2H), 5.43 (s, 2H), 3.21 (m, 6H), 2.00 (tt, *J* = 12.3 Hz, 1H), 1.72 (d, *J* = 11.2 Hz, 2H), 1.59 (d, *J* = 11.8 Hz, 2H), 1.48 (m, 1H), 1.27 (qd, *J* = 4.3 Hz, 2H), 0.86 (qd, *J* = 3.3 Hz, 2H). ESI-MS m/z (M+H)^+^ calculated for C_29_H_50_N_5_O_11_ 624.2, found 624.4; ESI-MS m/z (M+Na)^+^ calculated for C_29_H_29_N_5_NaO_11_ 646.2, found 646.4.

*Linker-MMAE conjugate* (**2**)

To a solution of **S17** (36.50 mg, 0.0585 mmol), MMAE (40.0 mg, 0.0557 mmol), and HOBt (8.0 mg, 0.0585 mmol) in DMF (3 mL) was added DIPEA (7.56 mg, 0.0585 mmol). The reaction mixture was stirred at rt overnight and then poured into water (20 mL). The mixture was extracted with ethyl acetate (3 × 20 mL), the organic layers were combined, washed with brine, dried over anhydrous Na_2_SO_4_, and evaporated under reduced pressure to give the crude product. Purification was performed by silica column chromatography in 80:1-10:1 DCM/methanol (v/v) to give 39.52 mg (59%) of **2** as a white solid. ^1^H-NMR (400 MHz, CDCl_3_): *δ* 8.29 - 7.98 (m, 2H), 7.62 - 7.50 (m, 3H), 7.37 - 7.30 (m, 4H), 7.28 - 7.23 (m, 1H), 6.69 (s, 2H), 6.64 - 6.53 (m, 2H), 5.64 - 5.46 (m, 0.5H), 5.37 - 5.10 (m, 1.5H), 4.96 - 4.91 (m, 1H), 4.73 - 4.61 (m, 2H), 4.23 - 3.99 (m, 4H), 3.84 - 3.69 (m, 1H), 3.62 - 3.28 (m, 14H), 2.97 - 2.83 (m, 5H), 2.47 - 2.21 (m, 3H), 2.11 - 2.02 (m, 3H), 1.89 - 1.68 (m, 9H), 1.43 - 1.23 (m, 9H), 1.02 - 0.67 (m, 24H). ESI-HRMS m/z (M+Na)^+^ calculated for C_62_H_91_N_9_NaO_15_ 1224.6532, found 1224.6522; ESI-MS m/z (M+2H)^2+^ calculated for C_62_H_93_N_9_O_15_ 601.8396, found 601.8398.

*2-amino-4-(hydroxymethyl)phenol* (**S19**)

To a solution of 4-hydroxy-3-nitrobenzaldehyde (550 mg, 3.3 mmol) in MeOH (10 mL) was added Pd/C (0.1g, cat), then stirred and hydrogen was introduced into the reaction flask. After 4h, the reaction mixture was filtered and the resultant filtrate was concentrated under reduced pressure. The residue was scattered in ether and the purification was performed by ultrasound and filtration 3 times to afforded 380 mg (84%) of the **S19** as a brown solid. ^1^H-NMR (400 MHz, DMSO-*d*_6_): *δ* 8.83 (s, 1H), 6.56-6.65 (m, 2H), 6.32 (dd, *J* = 7.8 Hz, 1H), 4.82 (t, *J* = 5.7 Hz, 1H,), 4.47 (br, 2H), 4.25 (d, *J* = 5.7 Hz, 2H). ESI-MS m/z (M+H)^+^ calculated for C_7_H_10_NO_2_ 140.1, found 140.2; ESI-MS m/z (M-H)^-^ calculated for C_7_H_8_NO_2_ 138.1, found 138.0.

*tert-butyl (2-((2-hydroxy-5-(hydroxymethyl)phenyl)amino)-2-oxoethyl)carbamate* (**S20**)

To a solution of *N*-Boc-glycine (400 mg, 2.28 mmol) in DMF (15 mL) was added EDCI (530 mg, 2.74 mmol), HOBt (370 mg, 2.74 mmol), DIPEA (440 mg, 3.42 mmol), and then stirred at rt for 90 min. Then, **S19** (320mg, 2.28 mmol) was added and stirred overnight. After completion of the reaction, the solution was poured into water (100mL), the aqueous layer was extracted with EtOAc (4 x 50 mL) and the combined organic layers was dried over Na_2_SO_4_ and concentrated. Purification was performed by silica column chromatography in 1:20 MeOH/DCM (v/v) to give 570 mg (84%) of **S20** as a orange solid. ^1^H-NMR (400 MHz, DMSO-*d*_6_): *δ* 9.81 (s, 1H), 8.98 (s, 1H), 7.92 (s, 1H), 7.32 (s, 1H), 6.85 (d, *J* = 8.2 Hz, 1H), 6.79 (d, *J* = 8.2 Hz, 1H,), 5.00 (br, 1H), 4.35 (s, 2H), 3.72 (s, 2H), 1.40 (s, 9H). ESI-MS m/z (M+H)^+^ calculated for C_14_H_21_N_2_O_5_ 297.1, found 297.2; ESI-MS m/z (M+Na)^+^ calculated for C_14_H_20_N_2_NaO_5_ 319.1, found 319.1.

*tert-butyl (2-((5-(hydroxymethyl)-2-((4-nitrobenzyl)oxy)phenyl)amino)-2-oxoethyl)carbamate* (**S21**)

To a solution of **S20** (0.15 g, 0.50 mmol) in DMF (5 mL) was added 1-(bromomethyl)-4-nitrobenzene (0.13 mg, 0.60 mmol) and CsCO_3_ (0.26 mg, 0.8 mmol). The reaction mixture was stirred at rt for 5 h. After the reaction ended, the reaction mixture was poured into water (25 mL) and the mixture was extracted with ethyl acetate (4 × 50 mL), the organic layers were combined, washed with brine, dried over anhydrous Na_2_SO_4_, and evaporated under reduced pressure to give the crude product. Further purification was performed by silica column chromatography to give 0.12 g (95%) of **S21** as a light yellow solid powder. ^1^H-NMR (400 MHz, DMSO-*d*_6_): *δ* 9.11 (s, 1H), 8.27 (d, *J* = 8.7 Hz, 2H), 8.02 (s, 1H), 7.77 (d, *J* = 8.7 Hz, 2H), 7.38 (s, 1H), 7.04 (d, *J* = 8.4 Hz, 1H), 6.98 (dd, *J* = 8.4 Hz, 1H), 5.36 (s, 2H), 4.39 (s, 2H), 3.76 (d, *J* = 5.9 Hz, 2H), 1.32 (s, 9H). ESI-MS m/z (M+H)^+^ calculated for C_21_H_26_N_3_O_7_ 432.2, found 432.2; ESI-MS m/z (M+Na)^+^ calculated for C_21_H_25_N_3_NaO_7_ 454.2, found 454.4.

*2-amino-N-(5-(hydroxymethyl)-2-((4-nitrobenzyl)oxy)phenyl)acetamide* (**S22**)

A solution of **S21** (150 mg, 0.35 mmol) in 2N HCl/EA (1 mL) was stired at rt for 2 h. The solvent was removed under reduced pressure to give the crude product, and purification was performed by beating with ethyl acetate to give 0.10 g (95% yield) of **S22** as a light pink powdery solid. ^1^H-NMR (400 MHz, DMSO-*d*_6_): *δ* 9.87 (s, 1H), 8.25 (t, *J* = 4.4 Hz, 2H), 7.79 (t, *J* = 8.6 Hz, 2H), 7.03 (m, 2H), 5.38 (s, 1H), 4.40 (s, 2H), 3.81 (m, 4H). ESI-MS m/z (M+H)^+^ calculated for C_16_H_18_N_3_O_5_ 332.12, found 332.12.

*6-(2,5-dioxo-2,5-dihydro-1H-pyrrol-1-yl)-N-(2-((5-(hydroxymethyl)-2-((4-nitrobenzyl)oxy)phenyl)amino)-2-oxoethyl)hexanamide* (**S23**)

A solution of **S22** (0.5 g, 1.36 mmol) in DMF (15 mL) was added **S4** (0.46 g, 1.5 mmol) and DIPEA (0.88 g, 6.8 mmol). The mixture was stired at rt overnight and then evaporated the solvent under reduced pressure to give a crude product, further purification was performed by silica column chromatography to give 0.23 g (43% yield) of **S23** as a reddish brown solid. ^1^H-NMR (400 MHz, DMSO-*d*_6_): *δ* 9.90 (s, 1H), 8.36 (t, 1H), 8.25 (d, *J* = 4.4 Hz, 2H), 7.98 (s, 1H), 7.78 (d, *J* = 8.7 Hz, 2H), 7.04 (d, *J* = 8.4 Hz, 1H), 7.01 (s, 2H), 6.98 (br, 2H), 5.34 (s, 2H), 5.12 (t, *J* = 5.8 Hz, 1H), 4.39 (d, *J* = 5.9 Hz, 2H), 3.89 (d, *J* = 5.6 Hz, 2H). 3.33 (t, *J* = 8.4 Hz, 2H), 2.06 (t, *J* = 7.6 Hz, 2H), 1.43 (m, 4H), 1.13 (m, 2H). ESI-MS m/z (M+H)^+^ calculated for C_26_H_29_N_4_O_8_ 525.2, found 525.5; ESI-MS m/z (M+Na)^+^ calculated for C_26_H_28_N_4_NaO_8_ 547.2, found 547.5.

*3-(2-(6-(2,5-dioxo-2,5-dihydro-1H-pyrrol-1-yl)hexanamido)acetamido)-4-((4-nitrobenzyl)oxy)benzyl (4-nitrophenyl) carbonate* (**S24**)

To a solution of **S23** (200 mg, 0.38 mmol) in DMF (10 mL) was added bis(*4*-Nitrophenyl) carbonate (230 mg, 0.75 mmol) and DIPEA (0.074 g, 0.57 mmol) and stirred at rt overnight. The reaction mixture was evaporated under reduced pressure and the purification was performed by silica column chromatography to give 165 mg (63%) of **S24** as a light yellow solid. ^1^H-NMR (400 MHz, DMSO-*d*_6_): *δ* 9.21 (s, 1H), 8.37 (t, *J* = 5.5 Hz, 1H), 8.31 (dt, *J* = 9.2 Hz, 2H), 8.27 (dt, *J* = 9.0 Hz, 2H), 8.15 (s, 1H), 7.79 (d, *J* = 8.7 Hz, 2H), 7.57 (dt, *J* = 9.2 Hz, 2H), 7.18 (dd, *J* = 8.4 Hz, 1H), 7.13 (d, *J* = 8.4 Hz, 1H), 6.98 (s, 2H), 5.40 (s, 2H), 5.22 (s, 2H), 3.91 (d, *J* = 5.6 Hz, 2H), 3.31 (m, 2H), 2.07 (t, *J* = 7.4 Hz, 2H), 1.43 (m, 4H), 1.14 (m, 2H). ESI-MS m/z (M+H)^+^ calculated for C_33_H_32_N_5_O_12_ 690.2, found 690.4; ESI-MS m/z (M+Na)^+^ calculated for C_33_H_31_N_5_NaO_12_ 712.2, found 712.5.

*Linker-MMAE conjugate* (**3**)

To a solution of **S24** (40.0 mg, 0.058 mmol), MMAE (40.0 mg, 0.057 mmol), and HOBt (0.90 mg, 0.07 mmol) in DMF (3 mL) was added DIPEA (13.57 mg, 0.11 mmol). The reaction mixture was stirred at rt overnight and then poured into water (20 mL). The mixture was extracted with ethyl acetate (3 × 20 mL), the organic layers were combined, washed with brine, dried over anhydrous Na_2_SO_4_, and evaporated under reduced pressure to give the crude product. Purification was performed by silica column chromatography in 80:1-10:1 DCM/methanol (v/v) to give 59 mg (80%) of **3** as a white solid. ^1^H-NMR (400 MHz, DMSO-*d*_6_): *δ* 9.15 (s, 1H), 8.37 - 8.24 (m, 3.5H), 8.09 - 8.06 (m, 1.5H), 7.90 (d, *J* = 9.2 Hz, 0.5H), 7.77 (d, *J* = 9.0 Hz, 2H), 7.65 (d, *J* = 9.2 Hz, 0.5H), 7.31 - 7.23 (m, 4H), 7.17 (t, *J* = 7.0 Hz, 1H), 7.06 (m, 2H), 6.99 (s, 2H), 5.43 (d, *J* = 5.3 Hz, 1H), 5.36 (s, 2H), 5.07 - 4.93 (m, 2H), 4.73 - 4.63 (d, 1H), 4.49 - 4.41 (m, 2H), 4.25 (m, 1H), 4.05 - 3.95 (m, 2H), 3.90 (d, *J* = 5.6 Hz, 2H), 3.79 (m, 1H), 3.57 - 3.47 (m, 1H), 3.31 (m, 2H), 3.24 - 3.16 (m, 7H), 3.11 (s, 2H), 3.05 - 2.96 (m,2H), 2.84 (m, 3H), 2.40 (d, *J* = 14.9 Hz, 2H), 2.26 (m, 1H), 2.13 - 2.04 (m, 4H), 1.75 (m, 3H), 1.56 - 1.33 (m, 6H), 1.23-0.96 (m, 11H), 0.88 - 0.72 (m, 17H). ESI-MS m/z (M+H)^+^ calculated for C_66_H_94_N_9_O_16_ 1268.6819, found 1268.6921; ESI-HRMS m/z (M+Na)^+^ calculated for C_66_H_93_N_9_NaO_16_ 1290.6638, found 1290.6636.

*(1r,4r)-4-((2,5-dioxo-2,5-dihydro-1H-pyrrol-1-yl)methyl)-N-(2-((5-(hydroxymethyl)-2-((4-nitrobenzyl)oxy)phenyl)amino)-2-oxoethyl)cyclohexane-1-carboxamide* (**S25**)

A solution of **S22** (1.87 g, 5.6 mmol) in DMF (40 mL) was added **S7** (2.06 g, 5.6 mmol) and DIPEA (2.17 g, 16.8 mmol). The mixture was stired at rt overnight and then evaporated the solvent under reduced pressure to give a crude product, further purification was performed by silica column chromatography to give 1.88 g (61% yield) of **S25** as a light yellow solid powder. ^1^H-NMR (400 MHz, DMSO-*d*_6_): *δ* 9.02 (s, 1H), 8.33 (t, *J* = 5.6 Hz, 1H), 8.25 (d, *J* = 8.7 Hz, 2H), 8.02 (s, 1H), 7.76 (d, *J* = 8.7 Hz, 2H), 7.03 (d, *J* = 8.4 Hz, 1H), 7.00 (s, 2H), 6.97 (dd, *J* = 8.1 Hz, 1H), 5.34 (s, 2H), 5.11 (t, *J* = 5.8 Hz, 1H), 4.38 (d, *J* = 5.6 Hz, 2H), 3.87 (d, *J* = 5.6 Hz, 2H). 3.19 (d, *J* = 7.0 Hz, 2H), 2.08 (tt, *J* = 12.1 Hz, 1H), 1.68 (d, *J* = 12.32 Hz, 2H), 1.55 (d, *J* = 12.9 Hz, 2H), 1.45 (m, 1H), 1.22 (qd, *J* = 12.3 Hz, 2H), 0.78 (qd, *J* = 12.8 Hz, 2H). ESI-MS m/z (M+H)^+^ calculated for C_28_H_31_N_4_O_8_ 551.2, found 551.5; ESI-MS m/z (M+Na)^+^ calculated for C_28_H_30_N_4_NaO_8_ 573.2, found 573.5.

*3-(2-((1r,4r)-4-((2,5-dioxo-2,5-dihydro-1H-pyrrol-1-yl)methyl)cyclohexane-1-carboxamido)acetamido)-4-((4-nitrobenzyl)oxy)benzyl (4-nitrophenyl) carbonate* (**S26**)

To a solution of **S25** (350 mg, 0.64 mmol) in DMF (10 mL) was added bis(*4*-Nitrophenyl) carbonate (580 mg, 1.90 mmol) and DIPEA (0.16 g, 1.27 mmol), and then stirred at rt overnight. The reaction mixture was evaporated under reduced pressure and the purification was performed by silica column chromatography to give 349 mg (76%) of **S26** as a light yellow solid. ^1^H-NMR (400 MHz, DMSO-*d*_6_): *δ* 9.14 (s, 1H), 8.31 (dt, *J* = 9.1 Hz, 2H), 8.27 (dt, *J* = 9.0 Hz, 2H), 8.19 (s, 1H), 7.78 (d, *J* = 8.7 Hz, 2H), 7.56 (dt, *J* = 9.2 Hz, 2H), 7.16 (m, 2H), 7.00 (s, 2H), 5.40 (s, 2H), 5.22 (s, 2H), 3.90 (d, *J* = 5.6 Hz, 2H), 3.19 (d, *J* = 7.0 Hz, 2H), 2.08 (tt, *J* = 12.0 Hz, 1H), 1.68 (d, *J* = 13.2 Hz, 2H), 1.53 (d, *J* = 12.9 Hz, 2H), 1.44 (m, 1H), 1.23 (m, 1H), 0.80 (qd, *J* = 12.3 Hz, 2H). ESI-MS m/z (M+H)^+^ calculated for C_35_H_34_N_5_O_12_ 716.2, found 716.4; ESI-MS m/z (M+Na)^+^ calculated for C_35_H_33_N_5_NaO_12_ 738.2, found 738.4.

*Linker-MMAE conjugate* (**4**)

To a solution of **S26** (66.0 mg, 0.092 mmol), MMAE (60.0 mg, 0.0836 mmol), and HOBt (11.35 mg, 0.084 mmol) in DMF (5 mL) was added DIPEA (16.0 mg, 0.125 mmol). he reaction mixture was stirred at rt overnight and then poured into water (20 mL). The mixture was extracted with ethyl acetate (3 × 20 mL), the organic layers were combined, washed with brine, dried over anhydrous Na_2_SO_4_, and evaporated under reduced pressure to give the crude product. Purification was performed by silica column chromatography in 80:1-10:1 DCM/methanol (v/v) to give 82.6 mg (69%) of **4** as a white solid. ^1^H-NMR (400 MHz, DMSO-*d*_6_): *δ* 9.12 (d, *J* = 15.9 Hz, 1H), 8.33 - 8.24 (m, 3.5H), 8.11 (m, 1.5H), 7.91 (d, *J* = 8.7 Hz, 0.5H), 7.76 (d, *J* = 8.7 Hz, 2H), 7.65 (d, *J* = 8.4 Hz, 0.5H), 7.31 - 7.24 (m, 4H), 7.16 (m, 1H), 7.05 (m, 2H), 7.00 (s, 2H), 5.43 (d, *J* = 5.3 Hz, 1H), 5.36 (s, 2H), 5.06 - 4.89 (m, 2H), 4.75 - 4.62 (d, 1H), 4.49 - 4.34 (m, 2H), 4.22 (m, 1H), 4.05 - 3.87 (m, 5H), 3.59 - 3.48 (m, 1H), 3.30 (m, 2H), 3.24 - 3.11(m, 9H), 3.05 - 2.97 (m, 2H), 2.87 - 2.81 (m, 3H), 2.43 (d, *J* = 16.8 Hz, 2H), 2.25 (m, 1H), 2.14 - 2.05 (m, 3H), 1.78 - 1.66 (m, 5H), 1.55 - 1.48 (m, 5H), 1.27 - 1.16 (m, 5H), 1.05-0.97 (m, 6H), 0.88 - 0.70 (m, 19H). ESI-MS m/z (M+H)^+^ calculated for C_68_H_96_N_9_O_16_ 1294.6975, found 1294.6976; ESI-HRMS m/z (M+Na)^+^ calculated for C_68_H_95_N_9_NaO_16_ 1316.6794, found 1316.6980.

*ethyl 2-amino-1-methyl-1H-imidazole-5-carboxylate* (**S27**)

Compound **S27** was synthesized as previously described.^10^ 50% over three steps yield as an orange solid; ^1^H-NMR (400 MHz, DMSO-*d_6_*): *δ* 7.27 (s, 1H), 6.19 (s, 2H), 4.15 (q, *J* = 7.1 Hz, 2H,), 3.52 (s, 3H), 1.23 (t, *J* = 7.1 Hz, 3H). ESI-MS m/z (M+H)^+^ calculated for C_7_H_12_N_3_O_2_ 170.1, found 170.3; ESI-MS m/z (M+Na)^+^ calculated for C_7_H_11_N_3_NaO_2_ 192.1, found 192.1.

*ethyl 1-methyl-2-nitro-1H-imidazole-5-carboxylate* (**S28**)

To a solution of sodium nitrite (600 mmol, 42 g) in water (110 mL) was added dropwise (over 45 min) a solution of aminoimidazole **S27** (50 mmol, 8.5 g) in acetic acid (70 mL) at -5 °C. The reaction mixture was stirred a further 30 min at -5 °C then warmed to rt and stirred for 16 h. The reaction mixture was diluted with DCM (100 mL) and the organic layer was separated. The aqueous layer was extracted with DCM (2 × 50 mL) and the combined organic layers was dried over Na_2_SO_4_ and concentrated. Purification was performed by silica column chromatography in 1:5 ethyl acetate/petroleum ether (v/v) to give 6.25 g (63%) of **S28** as a yellow solid. ^1^H-NMR (400 MHz, CDCl_3_): *δ* 7.75 (s, 1H), 4.40 (q, *J* = 7.0 Hz, 2H), 4.30 (s, 3H), 1.41 (t, *J* = 7.0 Hz, 3H). ESI-MS m/z (M+H)^+^ calculated for C_7_H_10_N_3_O_4_ 200.1, found 200.2; ESI-MS m/z (M+Na)^+^ calculated for C_7_H_9_N_3_NaO_4_ 222.0, found 222.1.

*1-methyl-2-nitro-1H-imidazole-5-carboxylic acid* (**S29**)

A mixture of **S28** (33 mmol, 6.6 g), and sodium hydroxide (100 mmol, 4 g) in water (125 mL) was stirred at rt overnight. The reaction mixture was acidified with concentrated HCl and the aqueous layer was extracted with EtOAc (3 x 100 mL). The combined organic layers was dried over Na_2_SO_4_ and concentrated to give 5.56 g (97%) of **S29** as a pale yellow solid. ^1^H-NMR (400 MHz, DMSO-*d*_6_): *δ* 13.83 (br, 1H), 7.74 (s, 1H), 4.18 (s, 1H). ESI-MS m/z (M-H)^-^ calculated for C_5_H_4_N_3_O_4_ 170.0, found 169.9; ESI-MS m/z (M+H)^+^ calculated for C_5_H_6_N_3_O_4_ 172.0, found 172.2.

*(1-methyl-2-nitro-1H-imidazol-5-yl)methanol* (**S30**)

To a cooled (-40 °C) solution of **S29** (5.8 mmol, 1 g) in anhydrous THF was added dropwise triethylamine (9.3 mmol, 1.3 mL) followed by isobutyl chloroformate (9.3 mmol, 1.2 mL) under argon. The reaction mixture was warmed to -10 °C and stirred for 1 h. Sodium borohydride (29 mmol, 1.1 g) was added to the reaction mixture followed by a mixture of THF:water (3:1) drop wise over 1 h. The reaction mixture was stirred for an additional 30 min at -10 °C then THF (30 mL) was added. The reaction mixture was filtered and the resultant filtrate was concentrated to give 0.76 g (69%) of **S30** as a yellow solid. ^1^H-NMR (400 MHz, DMSO-*d*_6_): *δ* 7.12 (s, 1H), 5.51 (t, *J* = 5.4Hz, 1H), 4.54 (d, *J* = 5.4Hz, 2H), 3.92 (s, 3H). ESI-MS m/z (M+H)^+^ calculated for C_5_H_8_N_3_O_3_ 158.1, found 158.4.

*5-(chloromethyl)-1-methyl-2-nitro-1H-imidazole* (**S31**)

To a solution of **S30** (500 mg, 3.2 mmol) in THF (10 mL) was added DIPEA (0.67 mL, 3.8 mmol) and mesyl chloride (0.3 mL, 3.8 mmol). The reaction was stirred at rt for 30 m. The reaction mixture was diluted with EtOAc (20 mL) and washed with 1N HCl (20 mL). The organic layer was separated, dried over Na_2_SO_4_ and concentrated. The crude product was scattered in ether and the purification was performed by ultrasound and filtration 3 times to afforded 450 mg (80.6%) of the **S31** as a yellow solid. ^1^H-NMR (400 MHz, DMSO-*d*_6_): *δ* 7.30 (s, 1H), 5.03 (s, 2H), 3.94 (s, 3H). ESI-MS m/z (M+H)^+^ calculated for C_5_H_7_ClN_3_O_2_ 176.0, found 176.2.

*tert-butyl(2-((5-(hydroxymethyl)-2-((1-methyl-2-nitro-1H-imidazol-5-yl)methoxy)phenyl)amino)-2-oxoethyl)carbamate* (**S32**)

To a solution of **S20** (100 mg, 0.34 mmol) in DMF (5 mL) was added 9 (70mg, 0.39 mmol) and CsCO_3_ (165 mg, 0.51 mmol). The reaction mixture was stirred at rt for 4 h then poured into water (40 mL), the mixture was extracted with EtOAc (4 x 50 mL) and the combined organic layers was dried over Na_2_SO_4_ and concentrated. Purification was performed by silica column chromatography in 1:20 MeOH/DCM (v/v) to give 120 mg (81%) of **S32** as a yellow solid. ^1^H-NMR (400 MHz, DMSO-*d*_6_): *δ* 8.92 (s, 1H), 8.01 (s, 1H), 7.37 (s, 1H), 7.32 (brt, *J* = 5.8 Hz, 1H,), 7.22 (d, *J* = 8.4 Hz, 1H), 7.03 (d, *J* = 8.4 Hz, 1H), 5.30 (s, 2H), 5.15 (t, *J* = 5.6 Hz, 1H), 4.41 (d, *J* = 5.6 Hz, 2H), 4.00 (s, 3H), 3.71 (d, *J* = 5.8 Hz,), 1.34 (s, 9H). ESI-MS m/z (M+H)^+^ calculated for C_19_H_26_N_5_O_7_ 436.2, found 436.4; ESI-MS m/z (M+Na)^+^ calculated for C_19_H_25_N_5_NaO_7_ 458.2, found 458.4.

*2-amino-N-(5-(hydroxymethyl)-2-((1-methyl-2-nitro-1H-imidazol-5-yl)methoxy)phenyl)acetamide* (**S33**)

To a solution of **S32** (1.44 g, 3.3 mmol) in DCM (10 mL) was added TFA (3.3mL). The reaction mixture was stirred at rt for 2 h, then evaporated under reduced pressure to give the crude product. The crude product **S33** is carried out directly without purification. ESI-MS m/z (M+H)^+^ calculated for C_14_H_18_N_5_O_5_ 336.1, found 336.2.

*6-(2,5-dioxo-2,5-dihydro-1H-pyrrol-1-yl)-N-(2-((5-(hydroxymethyl)-2-((1-methyl-2-nitro-1H-imidazol-5-yl)methoxy)phenyl)amino)-2-oxoethyl)hexanamide* (**S34**)

To a solution of **S33** (1.0 g, 2.2 mmol) in DMF (20 mL) was added DIPEA (1.0g, 1.4mL, 8 mmol) and stirred at rt for 30 min, then the **S4** (0.68g, 2.2 mmol)was added. The reaction mixture was stirred at rt overnight, and then evaporated under reduced pressure to give the crude product. The residue was dissolved in ethyl acetate and washed three times with water and saturated brine, respectively. The organic layers was dried over Na_2_SO_4_ and concentrated. Purification was performed by silica column chromatography in 1:20 MeOH/DCM (v/v) to give 850 mg (81%) of **S34** as a brown solid. ^1^H-NMR (400 MHz, DMSO-*d*_6_): *δ* 8.95 (s, 1H), 8.33 (t, *J* = 5.8 Hz, 1H,), 7.97 (s, 1H), 7.38 (s, 1H), 7.20 (d, *J* = 8.4 Hz, 1H), 7.03 (d, *J* = 8.4 Hz, 1H), 6.99 (s, 2H), 5.27 (s, 2H), 5.15 (t, *J* = 5.6 Hz, 1H), 4.40 (d, *J* = 5.6 Hz, 2H), 3.97 (s, 3H), 3.83 (d, *J* = 5.8 Hz, 2H), 3.34 (t, *J* = 7.3 Hz, 2H), 1.99 (t, *J* = 7.4 Hz, 2H), 1.49 - 1.37 (m, 4H), 1.18 - 1.11 (m, 2H). ESI-MS m/z (M+H)^+^ calculated for C_24_H_29_N_6_O_8_ 529.2, found 529.3; ESI-MS m/z (M+Na)^+^ calculated for C_24_H_28_N_6_NaO_8_ 551.2, found 551.3.

*3-(2-(6-(2,5-dioxo-2,5-dihydro-1H-pyrrol-1-yl)hexanamido)acetamido)-4-((1-methyl-2-nitro-1H-imidazol-5-yl)methoxy)benzyl (4-nitrophenyl) carbonate* (**S35**)

To a solution of **S34** (200 mg, 0.38 mmol) in DMF (10 mL) was added bis(*4*-Nitrophenyl) carbonate (230 mg, 0.75 mmol) and DIPEA (0.1 g, 0.75 mmol) and stirred at rt overnight. The reaction mixture was evaporated under reduced pressure and the purification was performed by silica column chromatography in 1:30 MeOH/DCM (v/v) to give 260 mg (97%) of **S35** as a light yellow solid. ^1^H-NMR (400 MHz, DMSO-*d*_6_): *δ* 9.06 (s, 1H), 8.35 - 8.30 (m, 3H), 8.16 (s, 1H), 7.57 (dt, *J* = 9.0 Hz, 2H), 7.40 (s, 1H), 7.30 (d, *J* = 8.7 Hz, 1H), 7.23 (dd, *J* = 8.4 Hz, 1H), 6.99 (s, 2H), 5.33 (s, 2H), 5.23(s, 2H), 3.99 (s, 3H), 3.86 (d, *J* = 5.6 Hz, 2H), 3.32 (t, *J* = 7.1 Hz, 2H), 2.00 (t, *J* = 7.4 Hz, 2H), 1.49-1.37 (m, 4H), 1.18-1.11 (m, 2H). ESI-MS m/z (M+H)^+^ calculated for C_31_H_32_N_7_O_12_ 694.2, found 694.2; ESI-MS m/z (M+Na)^+^ calculated for C_31_H_31_N_7_NaO_12_ 716.2, found 716.3.

*Linker-MMAE conjugate* (**5**)

To a solution of the **S35** (53 mg, 0.0766 mmol), MMAE (50 mg, 0.0696 mmol), HOBt (9.4 mg, 0.0696 mmol) in DMF (3 mL) was added DIPEA (13.5 mg, 18 μL, 0.1044 mmol). The mixture was stirred at rt overnight, and then poured into water (20 mL). The mixture was extracted with ethyl acetate (3 × 20 mL), the organic layers were combined, washed with brine, dried over anhydrous Na_2_SO_4_, and evaporated under reduced pressure to give the crude product. Purification was performed by silica column chromatography in 80:1-10:1 DCM/methanol (v/v) to give 66 mg (75%) of **5** as a white solid. ^1^H-NMR (400 MHz, DMSO-*d*_6_): *δ* 9.00 (s, 1H), 8.32 (t, *J* = 4.9 Hz, 1H), 8.10 - 8.08 (m, 1.5H), 7.91 (d, *J* = 9.8 Hz, 1H), 7.65 (d, *J* = 9.0 Hz, 0.5H), 7.38 (s, 1H), 7.32 - 7.22 (m, 5H), 7.19 - 7.08 (m, 2H), 6.99 (s, 2H), 5.43 (d, *J* = 5.0 Hz, 0.5H), 5.36 (d, *J* = 5.0 Hz, 0.5H), 5.29 (s, 2H), 5.14 - 4.90 (m, 2H), 4.75 - 4.62 (m, 1H), 4.49 - 4.35 (m, 2H), 4.26 (t, *J* = 10.0 Hz, 1H), 4.02 (t, *J* = 7.1 Hz, 2H), 3.97 (s, 3H), 3.84 (m, 2H), 3.56 (m, 1H), 3.46 (m,1H), 3.37 (m, 2H), 3.24 - 3.17 (m, 7H), 3.12 (s, 2H), 3.05 - 2.97 (m, 2H), 2.88 - 2.82 (m, 3H), 2.31 (d, *J* = 16.8 Hz, 1H), 2.25 (q, *J* = 10.1 Hz, 1H), 2.13 - 2.06 (m, 2H), 1.99 (t, *J* = 7.3 Hz, 3H), 1.82 - 1.68 (m, 3H), 1.53 - 1.37 (m, 6H), 1.33 - 1.11 (m, 5H), 1.05 - 0.97 (m, 6H), 0.92 - 0.75 (m, 17H). ESI-HRMS m/z (M+H)^+^ calculated for C_64_H_94_N_11_O_16_ 1272.6880, found 1272.6876; ESI-HRMS m/z (M+Na)^+^ calculated for C_64_H_93_N_11_NaO_16_ 1294.6699, found 1294.6697.

*Linker-MMAE conjugates* (**6**) and (**7**)

Compounds **6** and **7** were synthesized and characterized as we previously described.^11^

**Reference**

1 Wang, Y. et al. Antibody-Drug Conjugate Using Ionized Cys-Linker-MMAE as the Potent Payload Shows Optimal Therapeutic Safety. Cancers 12, 744, doi:10.3390/cancers12030744 (2020).

2 Wang, Y. *et al.* Novel Silyl Ether-Based Acid-Cleavable Antibody-MMAE Conjugates with Appropriate Stability and Efficacy. *Cancers* **11**, 957, doi:10.3390/cancers11070957 (2019).

3 Jeffrey, S. C. *et al.* Development and properties of beta-glucuronide linkers for monoclonal antibody-drug conjugates. *Bioconjug. Chem.* **17**, 831-840, doi:10.1021/bc0600214 (2006).

4 Sun, J., Hu, Z., Wang, R., Zhang, S. & Zhang, X. A Highly Sensitive Chemiluminescent Probe for Detecting Nitroreductase and Imaging in Living Animals. *Anal Chem* **91**, 1384-1390, doi:10.1021/acs.analchem.8b03955 (2019).

5 Zhou, L., Gong, L. & Hu, S. Construction of an efficient two-photon fluorescent probe for imaging nitroreductase in live cells and tissues. *Spectrochim Acta A Mol Biomol Spectrosc* **199**, 254-259, doi:10.1016/j.saa.2018.03.073 (2018).

6 Fang, Y., Shi, W., Hu, Y., Li, X. & Ma, H. A dual-function fluorescent probe for monitoring the degrees of hypoxia in living cells via the imaging of nitroreductase and adenosine triphosphate. *Chem Commun (Camb)* **54**, 5454-5457, doi:10.1039/c8cc02209g (2018).

7 Zhang, X., Zhao, Q., Li, Y., Duan, X. & Tang, Y. Multifunctional Probe Based on Cationic Conjugated Polymers for Nitroreductase-Related Analysis: Sensing, Hypoxia Diagnosis, and Imaging. *Anal Chem* **89**, 5503-5510, doi:10.1021/acs.analchem.7b00477 (2017).

8 Zhai, B. *et al.* A two-photon fluorescent probe for nitroreductase imaging in living cells, tissues and zebrafish under hypoxia conditions. *Analyst* **142**, 1545-1553, doi:10.1039/c7an00058h (2017).

9 Meng, F. *et al.* Molecular and cellular pharmacology of the hypoxia-activated prodrug TH-302. *Mol. Cancer. Ther.* **11**, 740-751, doi:10.1158/1535-7163.MCT-11-0634 (2012).

10 Karnthaler-Benbakka, C. *et al.* Targeting a Targeted Drug: An Approach Toward Hypoxia-Activatable Tyrosine Kinase Inhibitor Prodrugs. *ChemMedChem* **11**, 2410-2421, doi:10.1002/cmdc.201600417 (2016).

11 Wang, Y., Fan, S., Zhong, W., Zhou, X. & Li, S. Development and Properties of Valine-Alanine based Antibody-Drug Conjugates with Monomethyl Auristatin E as the Potent Payload. *International journal of molecular sciences* **18**, 1860, doi:10.3390/ijms18091860 (2017).
